# Synergistic antitumor interaction of valproic acid and simvastatin sensitizes prostate cancer to docetaxel by targeting CSCs compartment via YAP inhibition

**DOI:** 10.1186/s13046-020-01723-7

**Published:** 2020-10-08

**Authors:** Federica Iannelli, Maria Serena Roca, Rita Lombardi, Chiara Ciardiello, Laura Grumetti, Simona De Rienzo, Tania Moccia, Carlo Vitagliano, Angela Sorice, Susan Costantini, Maria Rita Milone, Biagio Pucci, Alessandra Leone, Elena Di Gennaro, Rita Mancini, Gennaro Ciliberto, Francesca Bruzzese, Alfredo Budillon

**Affiliations:** 1 Experimental Pharmacology Unit-Laboratory of Naples and Mercogliano (AV), Istituto Nazionale per lo Studio e la Cura dei Tumori “Fondazione G. Pascale” – IRCCS, Via M. Semmola, 80131 Naples, Italy; 2 Department of Clinical and Molecular Medicine, Sapienza University of Rome, Rome, Italy; 3 IRCCS “Regina Elena” National Cancer Institute, Rome, Italy; 4 Istituto Nazionale per lo Studio e la Cura dei Tumori “Fondazione G. Pascale” – IRCCS, Via Ammiraglio Bianco, 83013 Mercogliano, AV Italy

**Keywords:** Valproic acid, Statin, Mevalonate pathway, YAP, Prostate cancer, Cancer stem cells

## Abstract

**Background:**

Despite the introduction of several novel therapeutic approaches that improved survival, metastatic castration-resistant prostate cancer (mCRPC) remains an incurable disease. Herein we report the synergistic antitumor interaction between two well-known drugs used for years in clinical practice, the antiepileptic agent with histone deacetylase inhibitory activity valproic acid and the cholesterol lowering agent simvastatin, in mCRPC models.

**Methods:**

Synergistic anti-tumor effect was assessed on PC3, 22Rv1, DU145, DU145R80, LNCaP prostate cancer cell lines and EPN normal prostate epithelial cells, by calculating combination index (CI), caspase 3/7 activation and colony formation assays as well as on tumor spheroids and microtissues scored with luminescence 3D-cell viability assay. Cancer stem cells (CSC) compartment was studied evaluating specific markers by RT-PCR, western blotting and flow cytometry as well as by limiting dilution assay. Cholesterol content was evaluated by ^1^H-NMR. Overexpression of wild-type YAP and constitutively active YAP5SA were obtained by lipofectamine-based transfection and evaluated by immunofluorescence, western blotting and RT-PCR. 22Rv1 R_39 docetaxel resistant cells were selected by stepwise exposure to increasing drug concentrations. In vivo experiments were performed on xenograft models of DU145R80, 22Rv1 parental and docetaxel resistant cells, in athymic mice.

**Results:**

We demonstrated the capacity of the combined approach to target CSC compartment by a novel molecular mechanism based on the inhibition of YAP oncogene via concurrent modulation of mevalonate pathway and AMPK. Because both CSCs and YAP activation have been associated with chemo-resistance, we tested if the combined approach can potentiate docetaxel, a standard of care in mCRCP treatment. Indeed, we demonstrated, both in vitro and in vivo models, the ability of valproic acid/simvastatin combination to sensitize mCRPC cells to docetaxel and to revert docetaxel-resistance, by mevalonate pathway/YAP axis modulation.

**Conclusion:**

Overall, mCRPC progression and therapeutic resistance driven by CSCs via YAP, can be tackled by the combined repurposing of two generic and safe drugs, an approach that warrants further clinical development in this disease.

## Background

Prostate cancer (PCa) is the most commonly diagnosed male cancer in the developed world and a leading cause of cancer-related morbidity and mortality in men worldwide [1, 2]. Treatment of castration-resistant metastatic disease (mCRPC) with new-generation androgen-signaling inhibitors, has improved survival outcomes, however, mCRPC remains incurable and patients generally die within 2 years [3]. Docetaxel (DTX), the first chemotherapy approved for the treatment of mCRPC, remains a standard of care in this setting. Moreover, DTX was approved in metastatic or high-risk localized hormone-sensitive PCa in combination with androgen deprivation therapy [4]. However, systemic side effects hamper the patient’s compliance and DTX resistance invariably emerges, leading to disease relapse. Thus novel combination treatment strategies are needed to target signaling pathways involved in mCRPC progression and drug resistance.

Cancer stem cells (CSCs) actively contribute to the onset of chemo-resistance [5, 6] and their role in PCa has been shown [7]. A critical role of both metabolic as well as epigenetic reprogramming in the onset and maintenance of CSCs was demonstrated in several tumors, including PCa [8–10].

The mevalonate pathway (MVP) controls the biosynthesis of cholesterol, an essential component of mammalian cell membranes and precursor of steroid hormones, thus playing a critical role in PCa [11]. MVP provides also metabolites for post-translational protein prenylation such as farnesylation and geranyl-geranylation, which are critical for the downstream signaling activity of small GTPases such as Ras, Rho or Rac, heavily involved in tumor initiation and progression [12].

Statins, developed as lipid-lowering drugs, inhibit HMG-CoA reductase (HMGCR), the first step of the MVP, preventing cholesterol formation and the protein prenylation branch [13, 14]. Multiple epidemiological evidences suggested that statins could reduce risk, tumor aggressiveness, and mortality in PCa [15]. Moreover, a direct antitumor effect of statins in monotherapy [16–18] and in combination with both androgen-signaling inhibitors [17, 19] or DTX [20] has been shown.

Histone deacetylase inhibitors (HDACi) are an emerging family of anticancer agents that impair histone and non-histone proteins deacetylation, thus regulating different cancer altered pathways [21–24]. A large number of HDACi are currently in clinical development as anticancer agents, and four of them (vorinostat, belinostat, romidepsin and panobinostat) have been approved by the FDA [25–28]. Valproic acid (VPA), an approved anticonvulsant agent with histone deacetylase inhibitory activity and anticancer properties, has been investigated in cancer patients with a better safety profile compared with other HDACi [29].

In the present study, we suggest to repurpose VPA in combination with simvastatin (SIM), the most commonly used statin, as a novel antitumor approach for mCRPC treatment, by showing the efficacy of this combinatory approach to target the CSCs compartment, thus potentiating DTX antitumor effect and reverting DTX-resistance. Mechanistically we showed that VPA and SIM combination prevent the activity of the oncogene Yes-associated protein (YAP), a transcriptional regulator whose hyperactivation is an hallmark of several solid tumors, including PCa, being essential for cancer initiation/growth and drug-resistance [30].

## Methods

The drugs and their preparation, all other reagents including antibodies, probes, cell culture conditions, and other additional information are described in the Supplementary Methods.

### Cell proliferation assay and drugs combination studies

Cell proliferation was measured in 96-well plates in cells untreated and treated with VPA, SIM and DTX as single agent or in combination. Cell proliferation was measured using a spectrophotometric dye incorporation assay Sulforhodamine B [31]. Drugs combination studies were based on concentration-effect curves generated as a plot of the fraction of unaffected (surviving) cells versus drug concentration after 96 h of treatment. Synergism, additivity, and antagonism were quantified after an evaluation of the combination index (CI), which was calculated by the Chou-Talalay equation with CalcuSyn software (Biosoft, Cambridge, UK), as described elsewhere [32]. A CI < 0.9, CI = 0.9–1.2, and CI > 1.2 indicated a synergistic, additive or antagonistic effect, respectively. The dose reduction index (DRI) determines the magnitude of dose reduction allowed for each drug when given in combination, compared with the concentration of a single agent that is needed to achieve the same effect.

### Caspase 3/7 bioluminescence assay

The cells (5000 cells/well) were seeded into a 96-well plate and treated for 24 h with VPA, SIM and DTX alone or in combination. The combined caspase 3/7 activity was analyzed in triplicates using the Caspase-Glo® 3/7 Assay (Promega, Madison, WI, USA) according to the manufacturer’s protocol with some modifications. Briefly, after aspirating the medium, 50 μl of Caspase-Glo reagent and the samples were incubated at room temperature for 30 min. Subsequently, the caspase activities were assessed by measuring the luminescence in a Multilabel Reader VICTOR X4 2030 (PerkinElmer, Waltham, MA, USA).

### Flow cytometry analysis

To evaluate CD133 and CD44 surface expression 5 × 10^5^ cells were labeled with PE-conjugated anti-CD133 and FITC-conjugated anti-CD44 antibodies (see Supplementary Methods for antibodies details) for 15 min at 4 °C. Labeled cells were resuspended in Phosphate Buffer Saline (PBS)/0.5% Bovine Serum Albumine (BSA) and analyzed by FACScan flow cytometer (Becton Dickinson, Franklin Lakes, NJ, USA) acquiring 10,000 events for each sample.

Analysis of apoptosis by flow cytometry nuclear DNA staining by propidium iodide (PI) was performed by a FACScan flow cytometer (Becton Dickinson, Franklin Lakes, NJ, USA) acquiring 20,000 events for each sample. The percentage of apoptotic cells was calculated in the sub-diploid region of the DNA content, registered as FL2 signals in linear scale.

### Clonogenic agarassay

Cells were plated in 24-well, flat-bottomed plates using a two-layer soft agar system, as previously described [31]. After 3 h, the cells were treated with VPA and/or SIM at the in vitro IC_25_^96h^ of the drugs. The medium (with or without drugs) was replaced every 3 days. The colonies grew for 14 days and were then stained overnight with 3-(4,5-dimethylthiazolyl-2)-2,5-diphenyl tetrazolium bromide (MTT), photographed, analyzed, and counted using Image-Pro-Plus (Immagini and Computer, Bareggio, Milano, Italy). Colonies of > 100 mm were scored as positive.

### DTX-resistant cell selection

22Rv1 R_39 DTX-resistant cells were obtained by stepwise selection treating 22Rv1 with increasing doses of DTX (from 0.1 nM up to 6 nM) over 10 months. The selected cells were tested for drug resistance by evaluating the resistance index (RI) = IC_50_^96h^22Rv1 R_39/ IC_50_^96h^22Rv1.

### Protein extraction and western blotting

Cells grown and treated as indicated, were washed once with ice-cold PBS and centrifugated. The cell pellet was lyses by Nonidet P40 (Thermo Fisher Scientific, Waltham, MA USA) and clarified by centrifugation. Equal amount of protein, monitored by Bradford assay, was separed on 10% Sodium Dodecyl Phosphate (SDS) polyacrilamide gel electrophoresis (PAGE). Cytosol/membrane extract was obtained according to Baghirova S. et al. [33].

### Real-time PCR

Total RNA was isolated from cells, using Trizol® total RNA isolation reagent (Gibco, Gaitherburg, MD, USA), according to the manufacture’s recommendations. cDNA for qRT-PCR analyses was synthesized with the QuantiTect Reverse Transcription Kit (Qiagen, Valencia, CA, USA). mRNA expression levels were quantified by the fluorescent dye SYBR-green method (Qiagen, Valencia, CA, USA). Gene expression modulation was meseaured by the 2^−ΔΔCT^ method and normalized to β-actin levels as endogenous control.

### Spheroid-forming assay

Spheroids were cultured as described before [34] in Sphere Medium (DMEM/F12 supplemented with BSA, glucose, heparin, FGF, EGF, neuronal cell culure B27, insulin). The cells (40,000 cells/ml) were plated in low-attachment multiwell plates and treated with indicated drugs. Times and doses of treatments are described in results section. Spheroids were scored with CellTiter-Glo® 3D Cell Viability Assay (Promega, Madison, WI, USA).

### Plasmide transfection

Adherent 22Rv1 and 22Rv1 R_39 cells were transfected with YAP wild-type and YAP5SA plasmids as previously described by Noto A. et al. [35] using Lipofectamine 2000 Reagents (Invitrogen, Carlsbad, CA, USA), according to the manufacturer’s recommendation. After 48 h from transfection, cells were collected and western blotting, real-time PCR and immunofluorescent experiments were performed as described before.

### Immunofluorescence assay

Cells, plated on slides in 24-wells plate at 50000 cell/well, were treated with drugs as indicated in figure legends. Then cells were fixed in 4% paraformaldehyde (20 min at RT), blocked by 0.2% PBS/BSA solution (5 min at RT) and incubated with primary anti-YAP antibody for 1 h at 37 °C. After washes, cells were incubated with anti-rabbit Alexa Fluor 488 for 30 min at 37 °C and mounted on slide holder using mountant medium with 4′,6-diamidin-2-fenilindolo (DAPI) (Life technologies, Gaitherburg, MD, USA). Images were taken at 63X magnification by fluorescent microscope (AxioScope A1, Zeiss, Oberkochen, Germany).

### Evaluation of the cholesterol by ^1^H-nuclear magnetic resonance (NMR) spectroscopy

The cell pellets (2 × 10^6^ cells) and the tissues (100 mg) were subjected to a chemical extraction using methanol:water:chloroform (700 μL:520 μL:700 μL) as previously reported [36]. The apolar phases were collected, evaporated by SpeedVac system and was re-suspended in 700 μL of deuterated chloroform and trimethylsilylpropanoic acid. A 600-MHz Bruker Avance spectrometer equipped with a cryoprobe was used to acquire ^1^H spectra at 300 K for 256 scans. The spectral 0.50–6 ppm regions were integrated by the AMIX package in buckets normalized to the total spectrum area using Pareto scaling and Metaboanalyst tool [37]. We use as reference the proton signal of the cholesterol at 0.66 ppm because it was not overlapped with proton signals of other lipids. Significant differences between the proton signals of the cholesterol were evaluated by T-test and *p*-values < 0.05.

### Limiting-dilution assay

22Rv1 spheroid cultures were dissociated and live cells were FACS deposited using FACSaria (BD BiosciencesFranklin Lakes, NJ, USA) in a limiting dilution manner at 1, 2, 4, 8, 16, 32, 64 cells per well in ultra-low 96-well plates (Corning, NY, USA) in sphere medium. Stem cell frequency was evaluated after 3 weeks with the Extreme Limiting Dilution Analysis ‘limdil’ function as described by Colak S. et al. [38].

### In vivo xenograft studies

All studies have been performed in compliance with institutional guidelines and regulations (Directive 2010/63/EU; Italian Legislative Decree DLGS 26/2014) and after approval from the appropriate institutional review board (N.865/2015-PR). Five weeks old female NOD/SCID athymic mice (Charles River,Wilmington, MA, USA) were used for 22Rv1, 22Rv1 R_39 xenograft models and 4 weeks old female CD1 nude mice (Charles River,Wilmington, MA, USA) were used for DU145R80 xenograft model. Mice were acclimatized in the Animal Care Facility of Laboratory of Mercogliano (AV) Istituto Nazionale Tumori -“Fondazione G. Pascale” – IRCCS. After 1 week, cells (5 × 10^6^) diluted in 200 μl [PBS/Matrigel GF (Becton Dickinson) 1/1] were injected subcutaneously (s.c) in the flank regions of the mice. Based on pilot studies (data not shown), the mice were treated intraperitoneally (i.p.) with VPA (melted in water and diluted in a physiological solution) and SIM (melted in DMSO and diluited in physiological solution), plus DTX (melted in DMSO and diluited in physiological solution) once a week at the indicated concentrations. Mice in the control groups were treated with both physiological solution and/or DMSO plus physiological solution 1:1. Tumor volume (TV) (mm^3^), Tumor growth delay (TGD) and the percent change in the experimental groups was compared with that of the vehicle control groups as described before [29]. Tumor incidence curves to analyze tumor engraftment (first appearance of a palpable mass) was performed taking advantage of Kaplan-Meier approach.

### Statistical analysis

All experiments were performed at least three times. Statistical significance was determined by the one-way ANOVA, Tukey’s multiple comparison test, Dunn’s multiple comparisons test and Log Rank test; a *p* < 0.05 was considered to be statistically significant. All statistical evaluations were performed with GraphPad Prism 7.

## Results

### Valproic acid and simvastatin combination induces synergistic antitumor effect in prostate cancer cells via concurrent inhibition of the mevalonate pathway

We investigated the antitumor effect of VPA in combination with SIM in a panel of PCa cell lines (PC3, DU145, LNCaP, 22Rv1) with different molecular features. All cell lines resulted sensitive to the antiproliferative effects of both agents in monotherapy (Supplementary Table S1). DU145R80 cells, selected for resistance to the inhibitor of the prenylation arm of MVP zoledronic acid (ZOL) [39], were cross-resitant to SIM (RI of DU145R80 vs parental DU145 cells: 12.77) and sensitive to VPA. Then, we combined the two drugs, exploring different cytotoxic ratios, either equipotent doses (50:50 ratio) or one of the two drugs in excess (75:25 and 25:75 ratio) (Supplementary Table S2), and different treatment schedules, either simultaneously or sequentially (24 h delay between the two agents) (Supplementary Table S3).

**Fig. 1:**
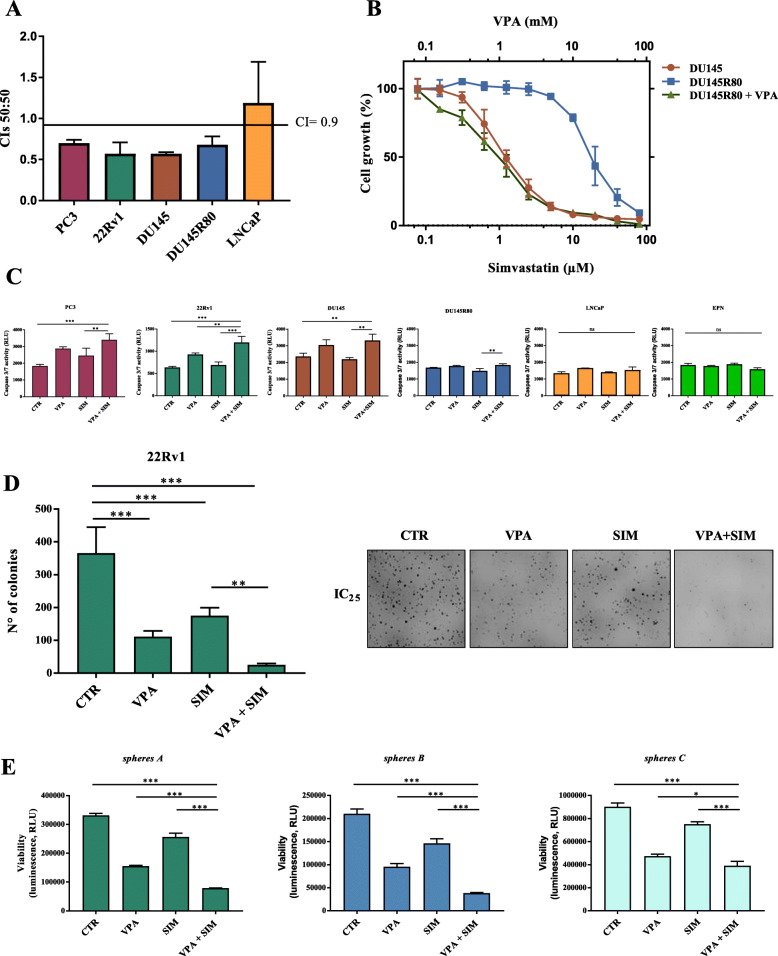
Combination of valproic acid and simvastatin induces antiproliferative and pro-apoptotic effect in 2D and 3D PCa models. **a** CI values (means ± SD from at least three separate experiments performed in quadruplicates) computed at 50% of cell kill (CI_50_) by CalcuSyn software after 96 h of treatment. Combinations were considered synergistic when CIs were below 0.9. **b** DU145R80 cells were treated for 96 h with increasing concentrations of SIM alone or with increasing doses of VPA and compared with DU145 treated with SIM alone. Cell growth expressed as percentage of control was assessed by sulforhodamine B colorimetric assay (see Methods). **c** Apoptosis, evaluated by Caspase 3/7 activity assay, in PC3, 22Rv1, DU145, DU145R80, LNCaP and EPN cells untreated or treated for 24 h with VPA and/or SIM at the respective IC_50_^96h^ doses (see Supplementary Table S1) and evaluated by luminescence assay. **d** Soft agar clonogenic assay of 22Rv1 cells, untreated or treated with VPA and SIM alone and in combination at 1 mM and 2,5 μM respectively (IC_25_^96h^ values). Colonies of > 100 μm were scored by a colony counter. Right: images from a representative experiment; left: values are the means ± S.D. from at least three independent experiments. **e** 22Rv1 cells (40,000/mL) were seeded in sphere medium in low attachment 96 multiwell. *Spheres A*: cells plated and concomitantly untreated or treated with VPA and/or SIM at the respective IC_50_^96h^ doses for 72 h. *Spheres B*: cells grown for 72 h then disaggregated and plated again in the presence of drugs for 72 h. *Spheres C*: spheres allowed to grow for 72 h and then treated for 72 h. Spheroids viability was assessed by luminescence assay. The values are the means ± S.D. from at least three independent experiments. Statistically significant results are reported (*** indicates *P* < 0.0005, ** indicates *P* < 0.005 and * indicates *P* < 0.05)

We obtained consistent antitumor synergistic effects with low CIs, calculated at 50% (CI_50_) of cell lethality, independently from the ratio of the two drugs used or the schedule tested, in all cell lines, except the LNCaP cells where an additive/antagonistic effect was observed (Fig. 1a; Supplementary Table S2). Interestingly, we also demonstrated that VPA treatment completely reverts SIM-resistance in DU145R80 cells, suggesting an impact of HDACi on MVP (Fig. 1b).

The synergistic antiproliferative effect induced by VPA/SIM correlated with a significant induction of apoptosis measured as caspase 3/7 activity after 24 h at IC_50_^96h^, with the exception of DU145R80 where only a slight pro-apoptotic effect was observed (Fig. 1c). Notably, in normal epithelial EPN cells we did not observe any pro-apoptotic effect of either agent or the combination, suggesting a selective action on tumor cells (Fig. 1c).

We also confirmed the synergistic antitumor effect of VPA/SIM combination in anchorage-independent condition on 22Rv1 (colony formation inhibition: VPA ∼ 58%; SIM ∼ 43%; VPA + SIM ∼ 86%) using low doses (IC_25_^96h^) (Fig. 1d), and similar data were obtained in DU145 and DU145R80 cells (Supplementary Fig. S1A and S1B).

Finally, to better recapitulate tumor growth complexity, we tested VPA/SIM combination also on PCa 3D-self-assembled spheroids. For these experiments we focused on 22Rv1 spheroids since this cell line resulted the most suitable for the growth in low attach condition using sphere medium compared with the other PCa cell lines (Fig. 1e and Supplementary Fig. S2A). We used different approaches to highlight different effects: (a) by evaluating treatments on 1st generation sphere formation (cells plated in low-attached plate in sphere medium and concomitantly treated), we investigated the capacity of treatment to prevent/reduce tumor formation (*spheres A*); (b) by treating 2nd generation sphere formation (cells were grown for 72 h, then disaggregated and plated again in the presence of drugs), we evaluated the impact of treatment to prevent/reduce more aggressive tumors (*spheres B*); (c) by treating formed-spheres (spheres allowed to grow for 72 h and then treated), we evaluated the capacity of treatment to induce tumor regression (*spheres C*). Our results showed that VPA/SIM combination, compared to single agents, strongly inhibits spheroid formation (spheres inhibition vs control: ∼76% in *spheres A*, ∼81% in *spheres B*), and induced ∼56% formed-sphere regression in *spheres C* vs control (Fig. 1e). Notably, compared to cell adhesion condition, 1st and 2nd generation spheres are normally described as enriched in CSC compartment [40–42] with self-renewal capacity. Indeed in both these 22Rv1 3D-models we showed the increased expression levels of CSC markers such as NANOg and OCT4 (Supplementary Fig. S2B-C) as well as CD44^+^ and CD133^+^ surface expression, compared to adherent cells (Supplementary Fig. S2D).

**Fig. 2:**
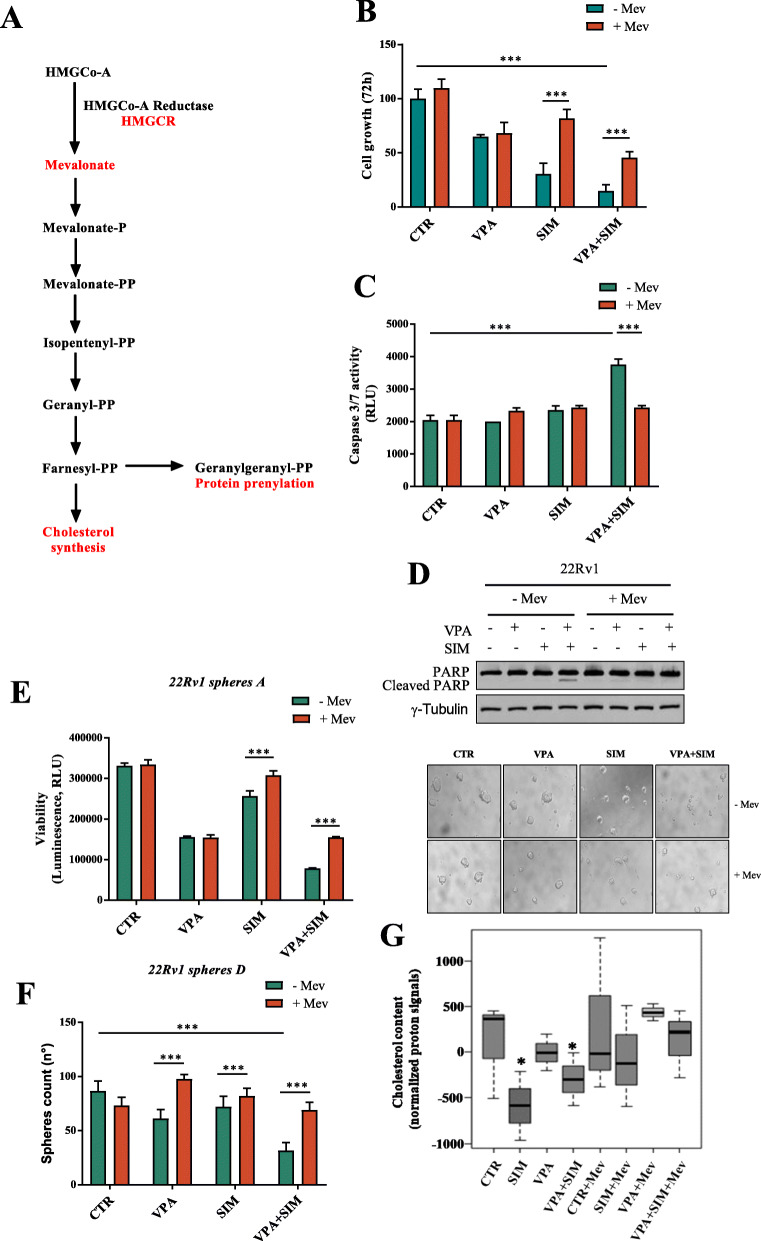
Mevalonic acid reverts the antiproliferative and apoptotic effect induced by valproic acid/simvastatin combination. **a** Overview of MVP and its principal inhibitors. **b** 22Rv1 cells untreated or treated for 72 h with VPA and/or SIM at the IC_50_^96 h^ doses ± Mev (100 μM) to bypass the inhibition of HMGCR. Cell growth expressed as percentage of control was assessed by sulforhodamine B colorimetric assay. The values, expressed as percentage of control, are the means ± S.D. from at least three independent experiments. **c** Apoptosis was evaluated by Caspase 3/7 activity assay in 22Rv1 cells untreated or treated for 24 h with VPA and/or SIM at the IC_50_^96 h^ doses ± Mev (100 μM). **d** Expression of cleaved PARP in 22Rv1 cell lines untreated or treated with VPA and/or SIM ± Mev (100 μM) for 24 h was evaluated by western blotting. γ-Tubulin was used as loading control. **e** 22Rv1 cells (40,000/mL) were seeded in sphere medium in low attachment 96 multiwell, to form 1st generation spheres (*spheres A)*, as indicated in Methods section, and treated with VPA and SIM at the IC_50_^96 h^ doses ± Mev (100 μM) for 72 h. Spheroids viability was assessed by luminescence assay. The values are the means ± S.D. from at least three independent experiments. Right: images from a representative experiment; left: values are the mean ± S.D. from at least three independent experiments. **f** 22Rv1 cells were seeded to form *spheres D*: cell seeded to form 1st generation spheres and concomitantly untreated or treated with VPA and /or SIM at the IC_50_^96 h^ doses ± Mev (100 μM) for 72 h, then disaggregated and plated again to form 2nd generation spheres without additional treatment for 72 h, and the spheroids were manually counted (left: values are the means ± S. D from at least three independent experiments). **g** Cholesterol content was measured by NMR spectroscopy in untreated or 22Rv1-treated as indicated at the IC_50_^96 h^ doses. The box and whisker plots summarize the normalized values of the proton signals of the cholesterol at 0.66 ppm for all samples. Statistically significant results are reported (*** indicates *P* < 0.0005, ** indicates *P* < 0.005 and * indicates *P* < 0.05)

To investigate whether the synergistic interaction between VPA and SIM occurred via MVP (schematically summarized in Fig. 2a) we evaluated the antitumor effect of the single agents or the combination, in the presence or absence of mevalonic acid (Mev), that overcomes the inhibition of HMGCR activity. Notably, the addition of Mev antagonized both the synergistic antiproliferative (Fig. 2b) and pro-apoptotic effect (Fig. 2c-d) induced by VPA/SIM combination on 22Rv1 cells grown in adherent condition or as *spheres A* (Fig. 2e).

To further evaluate the impact of treatment on putative CSCs, we analyzed the effect of the combination using an additional spheres growth system [34] (*spheres D* - Fig. 2f). In detail, 22Rv1 cells grown as spheroids were treated in 1st generation with VPA and SIM as single agents or in combination with or without Mev for 72 h; survived spheroids, were then disaggregated and plated again to form 2nd generation spheroids without additional treatment. Remarkably, a single VPA/SIM combination treatment in 1st generation, is able to affect 2nd generation spheroids formation (∼57% of inhibition vs control) and this effect was completely reverted by the addition of Mev (Fig. 2f).

Finally, as a readout of MVP inhibition we investigated the cholesterol content of 22Rv1 cell line in the different treatment setting, taking advantage of ^1^H-NMR metabolomic analysis of the cellular lipophilic (apolar) phase. As shown in Fig. 2g we observed a clear reduction of cholesterol content upon SIM treatment or in the combination setting and a slight reduction upon VPA treatment while all these effects were reverted by Mev.

Overall these data suggested that the synergistic interaction between VPA and SIM in PCa models could occur by targeting CSCs compartment via concurrent inhibition of the MVP.

### Valproic acid and simvastatin treatment targets CSCs compartment regulating YAP phosphorylation and nuclear localization in MVP-dependent manner

**Fig. 3:**
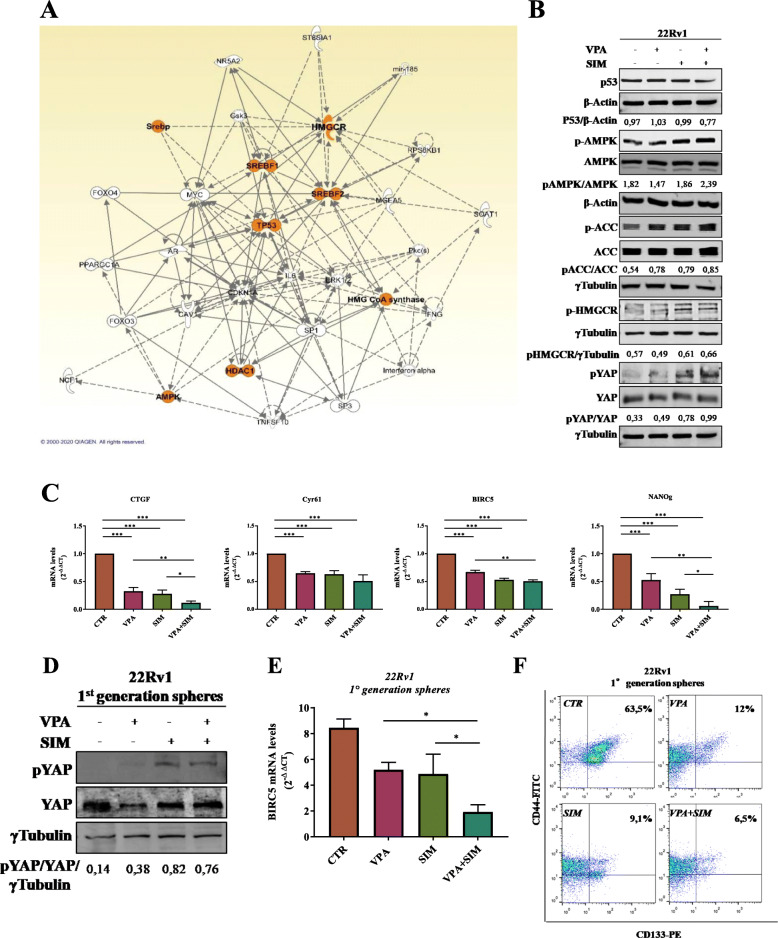
Valproic acid/simvastatin combination impairs YAP activity via modulation of mevalonate pathway and of AMPK. **a** The network was generated by Ingenuity Pathway Analysis (IPA) using “mevalonate pathway enzymes” and “HDAC inhibitors” search. Network genes are visualized by proper symbols, which specify the functional nature of the correspondent protein. Each node represents a gene and its direct (represented by solid lines) and indirect (represented by dotted lines) association with other genes. **b** Western blot analysis of p53, phospho AMPK (pAMPK), AMPK, phospho ACC (pACC), ACC, phosphoHMGCR (pHMGCR), phospho YAP (pYAP) and YAP in 22Rv1 treated with VPA and/or SIM at the IC_50_^96h^ doses for 4 h. Extracts (30 μg) were resolved by SDS-PAGE and immunoblotted using specific antibodies, βactin or γtubulin were used as loading control. Densitometric analysis was performed by ImageJ image software and reported as ratio relative to the indicated loading control. **c** CTGF, CYR61, BIRC5 and NANog mRNA expression was evaluated by RT-PCR after 4 h of cell culture in absence or presence of VPA and SIM at IC_50_^96h^ doses. β-actin was used as housekeeping control gene to normalize RT-PCR reactions. **d** Expression of phospho-YAP (pYAP) and YAP evaluated by western blotting in 22Rv1 1st generation spheres untreated or treated for 4 h with VPA, SIM or the combination at the IC_50_^96h^ doses. Γtubulin was used as loading control. Densitometric analysis was performed by ImageJ image software. **e** BIRC5 mRNA expression evaluated by RT-PCR in 22Rv1 1st generation spheres untreated or treated for 4 h with VPA, SIM or their combination at the IC_50_^96h^ doses. β-actin was used as housekeeping control gene to normalize RT-PCR reactions. **f** Surface marker expression (CD44 and CD113) was determined by flow cytometry on 22Rv1 1st generation spheres untreated or treated for 4 h with VPA, SIM or their combination at the IC_50_^96h^ doses. Statistically significant results are reported (*** indicates *P* < 0.0005, ** indicates *P* < 0.005 and * indicates *P* < 0.05)

To further disclose the molecular mechanism behind the synergistic antitumor interaction of VPA/SIM combination we performed an ingenuity pathway analysis (IPA) on “mevalonate pathway enzymes” and “HDAC inhibitors” combined search. As shown in Fig. 3a we revealed a network with direct and indirect relationships connecting HDAC1 and MVP enzymes (i.e. HMGCR, HMG-CoA synthase), as well as the transcription factors SREBF1 and 2 regulating MVP genes expression, all together confirming a functional relationship between the targets of our treatment combination. Indeed, we demonstrated the reciprocal ability of both VPA and SIM to target histone acetylation within 24 h (Supplementary Fig. S3A) and HMGCR mRNA expression within 2 h and up to 8 h of treatment at the IC_50_^96h^ (Supplementary Fig. S3B), in 22Rv1 cells. Moreover, at similar early time point (4 h), both VPA and SIM were able to reduce specifically HDAC1 and HDAC2 mRNA expression (Supplementary Fig. S3C), but not HDAC3 and HDAC6 (data not shown). Furthermore, upon VPA or SIM treatment we also showed the increase of RhoA cytoplasmatic and inactive form, that was reverted by either Mev or GGOH (Supplementary Fig. S3D), confirming the ability of both drugs to affect the MVP prenylation arm (see Fig. 2a).

However, the IPA network reported in Fig. 3a also highlighted additional hubs such as AMP-activate protein kinase (AMPK) or p53, both known regulators of the MVP. AMPK, a sensor of cellular energy status, is a known regulator of HMGCR activity [43, 44] and can be activated by both HDACi and statins [45]. We confirmed that the activating AMPK Tyr172 phosphorylation is induced by either VPA or SIM within 40 min (Supplementary Fig. S4A). Notably, this effect is paralleled by the induction of HMGCR inhibitory phosphorylation occurring within 2 h of VPA or SIM treatment (Supplementary Fig. S4A). Mutant p53 has been shown to trigger in cancer cells the MVP, leading to the aberrantly activation of YAP, an essential oncogene for cancer initiation/growth of most solid tumors, including PCa, an effect that can be reverted by statins [46]. Interestingly, recent reports also highlighted the ability of AMPK to regulate the Hippo pathway, directly inducing YAP inhibitory phosphorylation [47–49]. On these bases we evaluated YAP expression and activity in both p53 mutant 22Rv1 and p53 null PC3 cells upon VPA and/or SIM treatment. One of the most critical findings of our study was the clear synergistic induction of YAP inhibitory Ser127-phosphorylation induced by VPA/SIM combination within 4 h of treatment (Fig. 3b) and up to 24 h, (Supplementary Fig. S4B) paralleled by the synergistic activation of AMPK, its well-known downstream substrate acetyl-CoA carboxylase (ACC) and the inhibitory phosphorylation of HMGCR, in both 22Rv1 and PC3 cells (Fig. 3b, Supplementary Fig. S4C-D).

As a consequence of increased Ser127-phosphorylation induced by VPA/SIM combined treatment, YAP protein was retained in the cytoplasm (Supplementary Fig. S5A) and cannot translocate into the nucleus (Supplementary Fig. S5B). Indeed a clear reduction of YAP direct and indirect transcriptional targets CTGF, Cyr61, BIRC5 and NANOg [50–52] was observed upon VPA/SIM combined treatment (Fig. 3c). Notably, the inhibition of YAP activation was completely reverted by Mev which bypasses HMGCR inhibition, or GGOH, which bypasses prenylation arm inhibition (Supplementary Fig. S5A and S5B), thus confirming that YAP inhibition is dependent on VPA/SIM synergistic inhibition of MVP, at least in part via AMPK activation. Indeed, pharmacological inactivation of AMPK, with the specific inhibitor compound C, partially reverts the antiproliferative and apoptotic effect induced by VPA/SIM combination, both in PCa adherent cells and in 3D spheroids (Supplementary Fig. S6). Consistently with all the data reported above, the additional IPA network (Supplementary Fig. S5C), obtained by combining HMGCR and AMPK search, highlighted as hubs HDAC2 as well as CYR61, BIRC5 and CTGF YAP-transcriptional targets, further corroborating our results.

Interestingly compared to cell adhesion condition, 22Rv1 1st generation spheres showed an increased expression of YAP (Supplementary Fig.S2C) and one of its target gene CTGF (Supplementary Fig.S2E). We confirmed increased YAP inhibitory Ser127-phosphorylation induced by VPA, SIM and VPA/SIM combination in 22Rv1 3D spheroids (Fig. 3d) paralleled by a significant reduction of one of its target gene BIRC5 (Fig. 3e) and, most importantly, by the impairment of CSC CD44^+^/CD133^+^ surface markers (Fig. 3f).

Anyhow, the observed inhibition of YAP in our cell models is most likely p53-independent, because has been demonstrated in both 22Rv1 mut- and PC3 null-p53. Interestingly, LNCaP castration-sensitive wt-p53 cells, where we did not observe a VPA/SIM synergistic antitumor effect (Fig. 1a), expressed lower basal levels of AMPK, HMGCR and YAP (Supplementary Fig. S5D). Moreover, in this cell line we did not observe neither AMPK activation upon single agents or VPA/SIM combination, nor YAP increased inhibitory phosphorylation by combination treatment (Supplementary Fig. S5E). Furthermore, in this cell line combined VPA and SIM treatment significantly downregulated p53 levels (Supplementary Fig. S5E). Hence, the mechanism of the lack of VPA/SIM synergism in LNCaP cells require further investigation. Noteworthy, the three PCa cell lines where we reported a clear VPA/SIM synergistic antiproliferative and pro-apoptotic effect (PC3, 22Rv1, DU145), expressed high baseline protein levels of AMPK, HMGCR and YAP compared to EPN and LNCaP cells (Supplementary Fig. S5D), again suggesting that the mechanism by which VPA and SIM synergized requires the coordinated activation/addiction of/to these pathways. SIM-resistant DU145R80 cells, although expressed similar high levels of AMPK, showed reduced HMGCR and YAP basal protein levels, potentially related to the mechanism of SIM-resistance (Supplementary Fig. S5D).

**Fig. 4:**
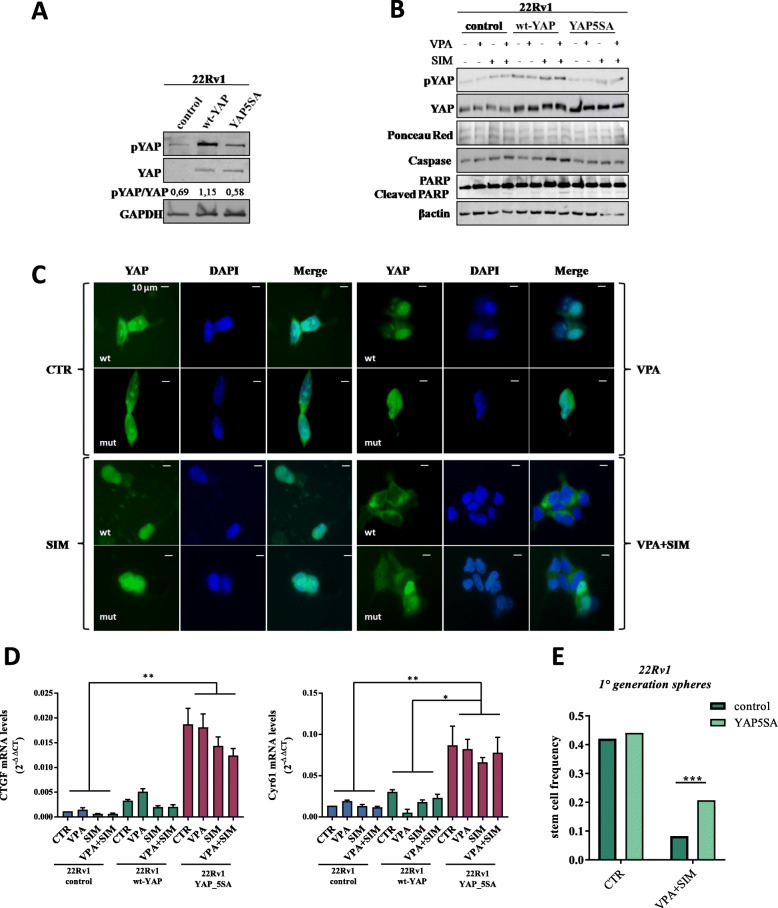
Constitutively active YAP5SA mutant reverts the effect of valproic acid and simvastatin combination. **a** Western blot analysis of phopho-YAP (pYAP), and YAP basal levels in control, wild-type YAP (wt-YAP)-transfected and YAP5SA (constitutively active)-transfected 22Rv1 cells. GAPDH serves as control for equal protein loading. Densitometric analysis was performed by ImageJ image software. **b** Western blot analysis of pYAP, YAP, Caspase 3 and PARP in 22Rv1 control, wt-YAP and YAP5SA cells, untreated or treated for 4 h with VPA and/or SIM at the IC_50_^96h^ doses. Βactin serves as control for equal protein loading. **c** YAP-immunofluorescence microscopy detection in 22Rv1 control, wt-YAP and YAP5SA cells untreated or treated indicated above. Cells were stained with anti-YAP antibody (green-Alexafluor488) and DAPI for nuclei detection (blue) and detected as indicated in Methods section. **d** CTGF and CYR61 mRNA expression evaluated by RT-PCR after 4 h of cell culture in absence or presence of VPA and SIM at the IC_50_^96h^ doses. The values are means ± S.D. of technical triplicates and Dunn’s multiple comparisons test was performed to compare the three groups for each genes. **e** Limiting dilution assay performed on 22Rv1 control and 22Rv1-YAP5SA cells, untreated or treated for 24 h with VPA/SIM at the IC_50_^96h^ doses and plated in ultra-low 96-well without additional treatment for three weeks. Clonal frequency was evaluated with the Extreme Limiting Dilution Analysis ‘limdil’ function as described in Methods section. Statistically significant results are reported (*** indicates *P* < 0.0005, ** indicates *P* < 0.005 and * indicates *P* < 0.05)

Next, in order to further confirm our observations, we evaluated the ability of VPA/SIM to affect YAP nuclear localization and activation, in 22Rv1 transiently transfected with either wild-type YAP (wt-YAP) or with the constitutively active mutated form YAP5SA [35] (Fig. 4a). As expected, we found that VPA/SIM were not capable to induce increased YAP-ser127 inhibitory phosphorylation as well as pro-apoptotic effect, as measured by increased Caspase 3 expression and PARP cleavage, in YAP5SA-transfected compared with non-trasfected or wt-YAP transfected 22Rv1 cells (Fig. 4b). Consequently, YAP nuclear translocation was not inhibited by the combined VPA/SIM treatment in YAP5SA-transfected compared with YAP wt-transfected cells, as shown by immunofluorescence experiments (Fig. 4c). Significantly a clear increased mRNA expression of CTGF and Cyr61, not signicantly changed upon treatments, was observed in YAP5SA-transfected cells compared with control non-transfected or wt-YAP transfected cells (Fig. 4d). Finally, to further investigate the effect of VPA/SIM combination on CSCs compartment in 22Rv1 control or YAP5SA-transfected cells we performed a limiting dilution assay, demonstrating a drammatic reduction in stem cell frequency induced by VPA/SIM combination (∼80% reduction), which was clearly lost in YAP5SA- transfected cells (∼43% of reduction), considering that the latter cell line also express endogenous wt-YAP (Fig. 4e).

All together, these data suggested that VPA/SIM combination induced antitumor effect in PCa models targeting CSCs compartment, via MVP-driven inhibition of YAP activation.

To explore the clinical relevance of YAP targeting in PCa we generated a signature of four genes induced by YAP (CTGF, CYR61, BIRC5 and ANRDK1) and interrogated the prostate adenocarcinoma Cancer Genome Atlas (TCGA). Notably, the four genes directly modulated by YAP were all highly enriched in the tumor tissues of relapsed PCa patients after curative resection compared with tumor free patients, suggesting a correlation of YAP activation with PCa prognosis and thus as potential drug target (Supplementary Fig. S7).

### Valproic acid and simvastatin combination sensitizes PCa cells to docetaxel treatment and reverts docetaxel-resistance by targeting CSCs compartment

**Fig. 5:**
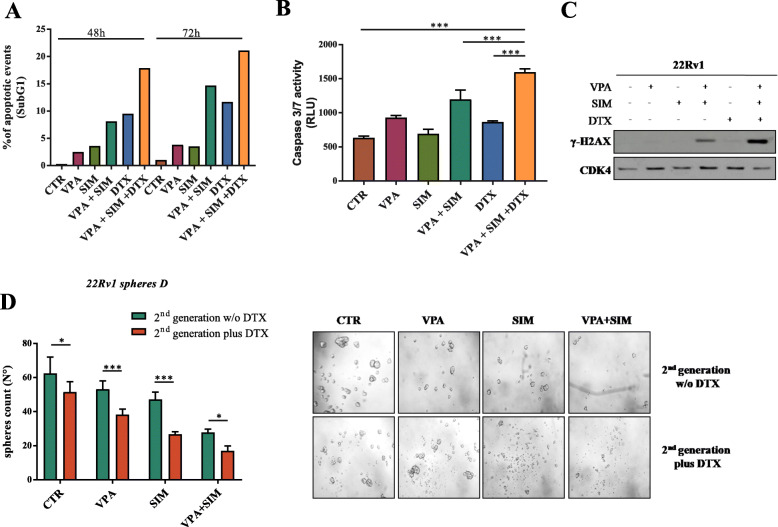
Valproic acid/simvastatin combination potentiate docetaxel antitumor effect in prostate cancer models. **a** 22Rv1 cells were treated or untreated with VPA, SIM and DTX at the respective IC_50_^96h^ doses for 48 and 72 h. Apoptotic effect was evaluated by flow cytometry as the percentage of hypodiploid population (sub-G0-G1). **b** Apoptosis was evaluated by Caspase 3/7 activity assay, in 22Rv1 cells untreated or treated as above and evaluated by luminescence assay. **c** Western blotting of γH2AX in 22Rv1 cells, untreated or treated as above; CDK4 serves as control for equal protein loading. **d** 22Rv1 cells (40,000 cells/mL) were seeded in sphere medium in low attachment 96 multiwell to form *spheres D*: cell seeded and concomitantly untreated or treated with VPA and SIM at the respective IC_50_^96h^, then disaggregated and plated again for 72 h in absence or presence of DTX at IC_50_^96h^ doses. The spheroids were counted and the values are the means ± S. D from at least three independent experiments (Right panel: representative images from spheres D experiment in E). Statistically significant results are reported (*** indicates *P* < 0.0005, ** indicates *P* < 0.005 and * indicates *P* < 0.05)

Several evidences suggest that the CSCs compartment critically contributes to chemoresistance. Thus, we next explore the potential of VPA/SIM combination to sensitize PCa cells to DTX. We investigated the triple combination of VPA, SIM and DTX at equitoxic concentrations either simultaneously or sequentially (with a 24 h delay between concomitant VPA/SIM and DTX or vice versa) (Table 1). We obtained consistent synergistic anti-proliferative effects of the triple combination VPA/SIM/DTX with the lowest CI_50_ values in all the four PCa cell lines tested, compared with dual combinations (VPA/DTX or SIM/DTX), and independently of the schedule used. The clear potentiation of DTX cytotoxic effect by VPA/SIM combination was also confirmed by the dose reduction indexes (DRIs), the order of magnitude (fold) of dose reduction obtained for the IC_50_ (DRI_50_) in combination vs single drug treatments, which ranged for DTX, among the cell lines tested, from 4 up to 20-fold, in triple combination treatment (Table 1). Furthemore, we also showed a synergistic induction of apoptosis in 22Rv1 (Fig. 5a-b), DU145 and PC3 cells (Supplementary Fig. S8A-B), and of DNA damage assessed as H2AX phosphorylation (γH2AX) in 22Rv1 cells (Fig. 5c), by the triple VPA/SIM/DTX combination vs single agents or dual combinations. Notably, in EPN normal epithelial cells, we did not potentiate the pro-apototic effect of DTX in either dual or triple combination treatments (Supplementary Fig. S8A-B).

**Table 1:** Combination index (CI) and dose reduction index (DRI) values for valproic acid (VPA), simvastatin (SIM) and docetaxel (DTX) combinations accordingly to different treatment schedules

Cell Lines	VPA + DTX	SIM + DTX	VPA + SIM + DTX	VPA + SIM→DTX	DTX→ VPA + SIM
**PC3**	^a^CI_50_: 0.930.05 ^b^DRI_50_VPA:1.4 ± 0.07 DRI_50_ DTX: 4.7 ± 0.94	CI_50_: 0.95 ± 0.06 DRI_50_SIM:1.42 ± 0.13 DRI_50_ DTX:4.8 ± 2.8	CI_50_: 0.71 ± 0.06 DRI_50_ VPA:3.1 ± 0.05 DRI_50_ SIM:3.8 ± 1.7 DRI_50_ DTX: 10.9 ± 2.55	CI_50_: 0.62 ± 0.17 DRI_50_ VPA:4.06 ± 1.89 DRI_50_ SIM:3.49 ± 1.0 DRI_50_ DTX: 18.4 ± 4.4	CI_50_: 0.54 ± 0.06 DRI_50_ VPA: 5.3 ± 0.6 DRI_50_ SIM: 3.9 ± 0.5 DRI_50_ DTX: 10.2 ± 2.76
**22Rv1**	CI_50_: 0.7 ± 0.09 DRI_50_ VPA: 2.3 ± 0.55 DRI_50_ DTX: 3.07 ± 0.9	CI_50_: 0.55 ± 0.13 DRI_50_ SIM:2.9 ± 0.4 DRI_50_DTX:4.2 ± 1.75	CI_50_: 0.48 ± 0.1 DRI_50_ VPA:6.0 ± 1.5 DRI_50_ SIM:5.4 ± 1.9 DRI_50_ DTX: 7.3 ± 0.22	CI_50_: 0.55 ± 0.13 DRI_50_ VPA:2.3 ± 0.5 DRI_50_ SIM:4.05 ± 1.82 DRI_50_ DTX: 4.05 ± 1.82	CI_50_: 0.48 ± 0.1 DRI_50_ VPA: 6.0 ± 1.5 DRI_50_ SIM: 5.4 ± 1.9 DRI_50_ DTX: 9.8 ± 4.3
**DU145**	CI_50_: 0.87 ± 0.14 DRI_50_ VPA:1.9 ± 0.2 DRI_50_ DTX: 3.9 ± 0.5	CI_50_: 0.86 ± 0.2 DRI_50_SIM:2.42 ± 01.2 DRI_50_DTX:2.54 ± 0.2	CI_50_: 0.65 ± 0.03 DRI_50_ VPA:2.84 ± 0.1 DRI_50_ SIM:6.46 ± 2.3 DRI_50_ DTX: 6.99 ± 0.6	CI_50_: 0.64 ± 0.04 DRI_50_ VPA:0.45 ± 0.07 DRI_50_ SIM:5.65 ± 2.05 DRI_50_ DTX:18.3 ± 0.03	CI_50_: 0.84 ± 0.02 DRI_50_ VPA: 2.32 ± 0.03 DRI_50_ SIM: 5.35 ± 0.07 DRI_50_ DTX: 4.33 ± 0.38
**DUR80**	CI_50_: 0.67 ± 0.15 DRI_50_ VPA:1.9 ± 0.6 DRI_50_ DTX: 6.59 ± 0.8	CI_50_: 0.54 ± 0.2 DRI_50_SIM:53.9 ± 14.5 DRI_50_DTX:2.02 ± 0.7	CI_50_: 0.67 ± 0.02 DRI_50_ VPA:2.02 ± 0.2 DRI_50_ SIM:194.5 ± 20 DRI_50_ DTX: 7.3 ± 1.9	CI_50_: 0.51 ± 0.25 DRI_50_ VPA:2.5 ± 1.4 DRI_50_ SIM:216.1 ± 0.07 DRI_50_ DTX:19.9 ± 0.05	CI_50_: 0.65 ± 0.02 DRI_50_ VPA: 2.21 ± 0.01 DRI_50_ SIM: 179.6 ± 0.14 DRI_50_ DTX: 4.49 ± 0.62

Cell growth assessment was done by sulforhodamine B colorimetric assay (see Methods). ^a^CIs values (mean ± S.D.) from at least three separate experiments performed in quadruplicate) computed at 50% of cell kill (CI_50_) by CalcuSyn software (Biosoft,Cam- bridge, UK). CIs smaller than 0.8 indicate strong synergism; CIs smaller than 0.9 indicate sinergysm; additivity between 0.9 and 1.2 or antagonism more than. Equipotent doses (50:50 cytotoxic ratio) of each of the two agents were evaluated after 96 h with a simultaneous (VPA + DTX: SIM + DTX or VPA + SIM + DTX) or sequential exposure with 24 h delay to either drug (VPA + SIM → DTX; DTX → VPA + SIM) as described in Methods section

^b^DRI values (mean ± S.D.) from at least three separate experiments performed in quadruplicate) represent the order of magnitude (fold) of dose reduction obtained for IC_50_ (DRI_50_) in combination setting compared with each drug alone

The effect of the triple combination was further investigated in two different 3D 22Rv1 cell colture systems such as hanging-drop microtissues (Supplementary Fig. S8C-D) and self-assembled spheroids (Supplementary Fig. S8E). We confirmed a strong inhibitory effect of VPA/SIM combination on both microtissues or spheres formation and regression but we also observed a potentiation of DTX effect, which, as single agent, was poorly effective. More importantly, in *sphere D* system, the pre-treatment with VPA/SIM combination of 22Rv1 cells during the 1st generation of spheroids formation, strongly potentiated the efficacy of DTX given alone to surviving cells during 2nd generation of spheroid formation, sustaining again the hypothesis that VPA/SIM combination, is able to sensitize PCa tumorsphere to DTX treatment by targeting the CSCs compartment (Fig. 5d).

**Fig. 6:**
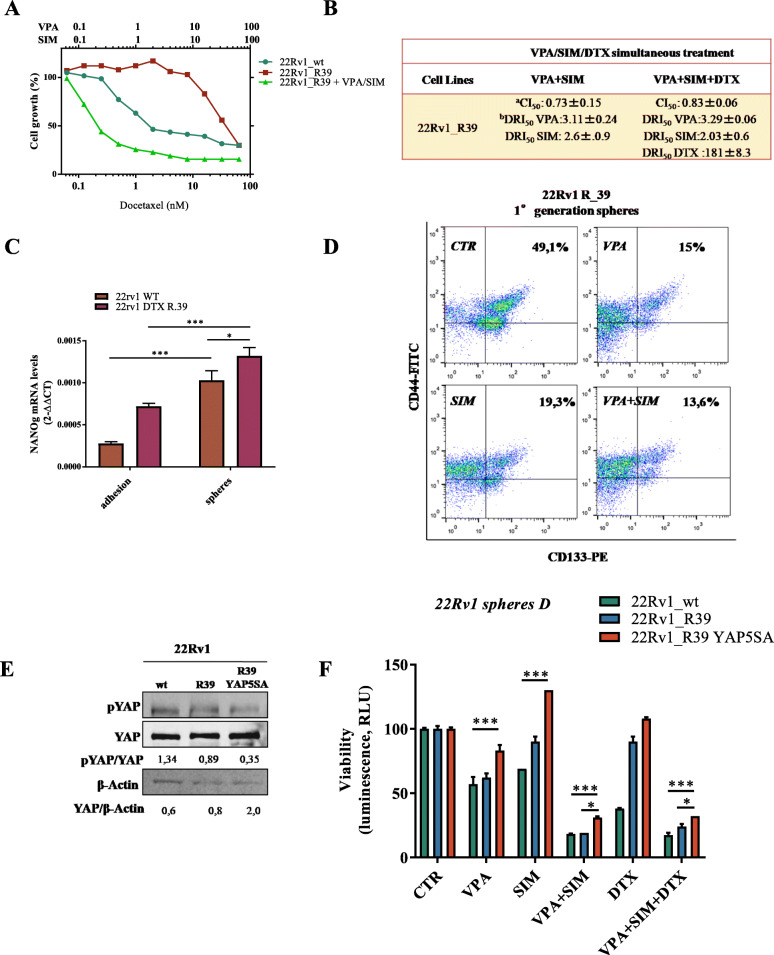
Valproic acid/simvastatin combination reverts docetaxel-resistance in prostate cancer models. **a** Dose response curves of 22Rv1 R_39 cells treated for 96 h with increasing concentrations of DTX alone or with VPA/SIM combination and compared with 22Rv1_wt treated with DTX alone. Cell growth is expressed as percentage of control and was assessed by sulforhodamine B colorimetric assay (see Methods). **b** Combination Index (CI) and Dose Reduction Index (DRI) values (means ±S.D.) of VPA/SIM or VPA/SIM/DTX combinations (50:50 cytotoxic ratio) at 96 h in 22Rv1 R_39 cells from at least three separate experiments performed in quadruplicate computed at 50% of cell kill (CI_50_) by CalcuSyn software (Biosoft,Cam- bridge, UK). ^a^CIs: < 0.8 strong synergism; CIs < 0.9 sinergysm; between 0.9 and 1.2 additivity; (or > 1.2 antagonism. ^b^DRI values represent the order of magnitude (fold) of dose reduction obtained for IC_50_ (DRI_50_) in combination setting compared with each drug alone. **c** NANOg mRNA expression evaluated by RT-PCR at basal level in both 22Rv1 wt and 22Rv1 R_39 in adhesion conditions and in 1st generation spheres. β-actin was used as housekeeping control gene to normalize RT-PCR reactions. **d** Surface marker expression (CD44 and CD113) was determined by flow cytometry on 22Rv1 R_39 cells treated for 24 h with VPA and/ SIM at the IC_50_^96h^ doses. **e** Western blot analysis of basal pYAP, and YAP, in 22Rv1 (wt), 22Rv1 R_39 and YAP5SA-tansfected 22Rv1 R_39 cells. Densitometric analysis was performed by ImageJ image software; ponceau red serves as loading control. **f** 22Rv1_wt, 22Rv1 R_39 and YAP5SA –transfected 22Rv1 R_39 2nd generation spheres (sphereD). Cells (40,000/mL) were seeded in sphere medium in low attachment 96 multiwell untreated or treated with VPA and/or SIM and/or DTX at the IC_50_^96h^ doses relative to 22Rv1 wt cells, then disaggregated and plated again for 72 h without additional treatment. Spheroids viability was assessed by ATP luminescence assay. The values are the means ± S.D. from at least three independent experiments. Statistically significant results are reported (*** indicates *P* < 0.0005, ** indicates *P* < 0.005 and * indicates *P* < 0.05)

Consistently with the latest results, taking advantage of the DTX-resistant 22Rv1cells (22Rv1_ R39), generated in our lab by stepwise incresing concentrations of DTX (DTX resistance index vs parental 22Rv1 cells: 39.5), we also demonstrated that VPA/SIM combination was able to revert DTX resistance (Fig. 6a). Indeed we confirmed a clear synergism in triple combination (VPA/SIM/DTX), with a CI_50_ smaller then 0.9 and DRI_50_ for DTX of up to 180 fold in DTX-resistant 22Rv1_R39 cells (Fig. 6b). Notably, compared to 22Rv1 parental cells 22Rv1 R_39 showed an increased basal level expression of NANOg mRNA both in adhesion and 1st generation spheres conditions (Fig. 6c) and reduced basal YAP-ser127 inhibitory phosphorylation (Fig. 6e). Moreover VPA and/or SIM treatment clearly reduced CD44^+^/CD133^+^ subpopulation in 22Rv1 R_39 grown as CSC enriched 1st generation spheroids (Fig. 6d, Supplemetary Fig.S9A). Consistently, by using the *sphere D* system, we confirmed VPA/SIM ability to revert DTX resistance on pre-treated tumorsphere, but this effect was partially loss in 22Rv1 cells transfected with mutated YAP (< 79% vs < 43% of spheroid formation reduction in 22Rv1 cells and 22Rv1-YAP5SA, respectively) (Fig. 6e-f; Supplemetary Fig. 9B).

Altogether these evidences demonstratd that VPA/SIM combination potentiate the efficacy of the chemotherapeutic DTX and revert DTX-resistance, by targeting CSCs compartments via YAP-activity inhibition.

### In vivo synergistic antitumor effect of valproic acid and simvastatin in combination with docetaxel

**Fig. 7:**
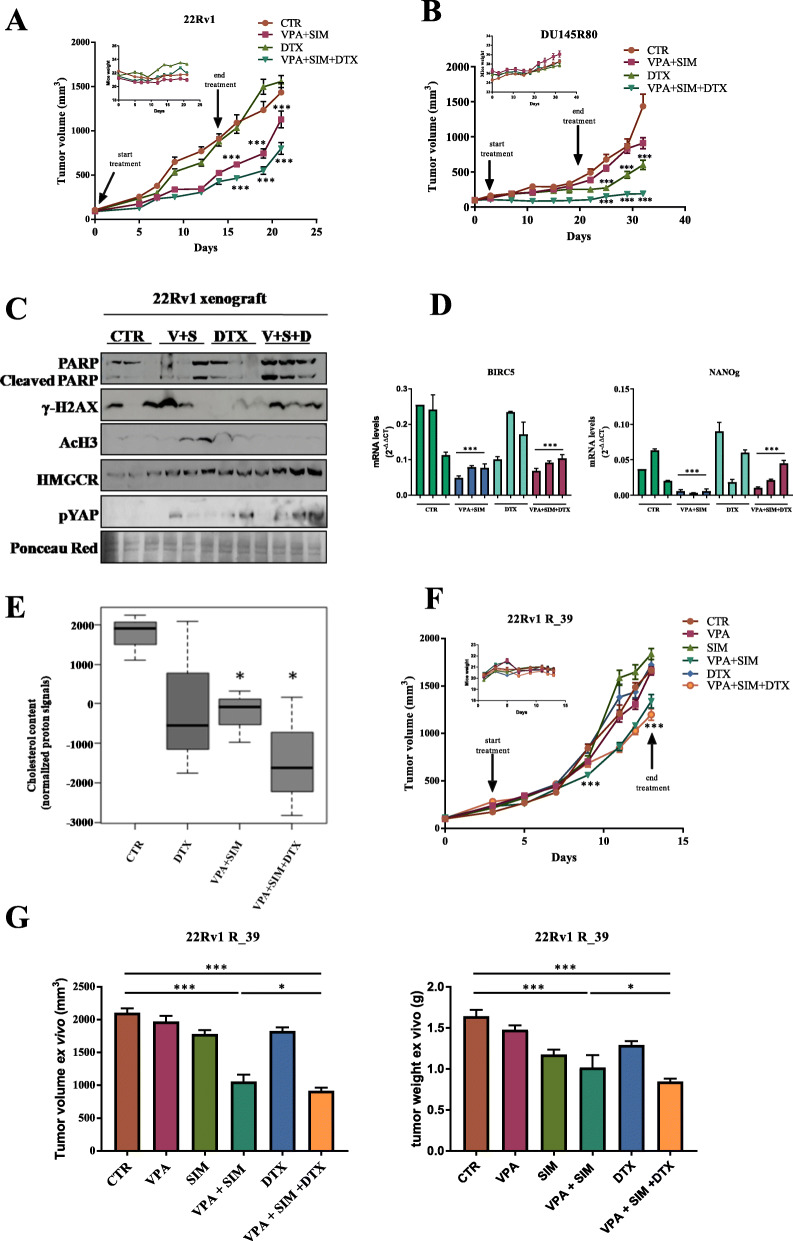
Valproic acid/simvastatin combination potentiates docetaxel antitumor effect and reverts docetaxel-resistance in vivo in prostate cancer xenograft models. **a** 22Rv1 and **b** DU145R80 cells (5 × 10^6^), were s.c. injected into athymic mice as described in the Methods section. When established tumors were palpable, mice were treated with vehicles or VPA (200 mg/kg i.p.) and SIM (2 mg/Kg i.p.) combination every day for two weeks, DTX (10 mg/Kg i.p.), once a week, or triple VPA/SIM/DTX combination. Relative tumor volume curves for 22Rv1 (left panel) and DU145R80 (right panel) xenografts. Means ± SD tumour volume measured at pre-specified time points. *Inset*, body weight measured two times/week. **c** Expression of cleaved PARP, γH2AX, AcH3, HMGCR and pYAP protein expression from xenograft 22Rv1 tumor samples evaluated by western blot (abbreviation = V + S: VPA + SIM; V + S + D: VPA + SIM + DTX); ponceau red was used as loading control. **d** BIRC5 (left panel) and NANOg (right panel) mRNA expression determined by RT-PCR in 22Rv1 samples. β-actin was used as housekeeping control gene to normalize RT-PCR reactions. Tukey’s multiple comparisons test, demonstrated statistically significant diffrences for VPA/SIM and VPA/SIM/DTX groups versus CTR and DTX groups. **e** Cholesterol content measured by NMR spectroscopy in tumor samples from untreated or 22Rv1-treated xenografts as indicated. The box and whisker plots summarize the normalized values of the proton signals of the cholesterol at 0.66 ppm for all samples. **f** 22Rv1 R_39 cells (6 × 10^6^) were s.c. injected into athymic mice as described in the Methods section. When established tumors were palpable, mice were treated with vehicles or VPA and/or SIM every day for two weeks, and or DTX once a week, or triple VPA/SIM/DTX combination at the dosages indicated above for 22Rv1 parental cells xenograft model. Means ± SD tumor volume measured at pre-specified time points. *Inset*, body weight measured two times/week. **g** Ex vivo volume (left panel) and weight (right panel) of tumors collected at the end of the experiment (day 13). Statistically significant results are reported (*** indicates *P* < 0.0005, ** indicates *P* < 0.005 and * indicates *P* < 0.05)

Finally, to assess whether the synergistic antitumor effects demonstrated in vitro could be confirmed in vivo we evaluated VPA and SIM in combination with DTX in both 22Rv1 and DU145R80 xenograft models as well as in 22Rv1 R_39 DTX resistant cells. This was accomplished through the measurement of tumor volume (Fig. 7a-b and f), the percent of tumor volume change and the tumor growth delay (TGD) (Supplemetary Fig. S10A-C). Specifically, twenty-eight mice were injected with either 22Rv1 or DU145R80 cells, and randomly assigned to four groups to receive DTX (10 mg/Kg i.p weekly for 2 weeks), VPA/SIM combination (200 mg/Kg and 2 mg/Kg, respectively, i.p. daily for 2 weeks), the triple combination, or their vehicles. Notably, the dosages of DTX, VPA and SIM correspond or were lower to those reported in the literature for in vivo experiments [32, 53, 54], and were consistent with corresponding doses used in patients [32, 54, 55].

As shown in Fig. 7a vehicles-treated 22Rv1 xenografted tumors grew rapidly and reached the endpoint size within 3 weeks; at this time-point DTX was not effective, VPA/SIM combination produced a clear statistically significant tumor growth inhibition compared with control mice, while triple combination treatment induced a further significant inhibition of tumor growth. Notably, in SIM-resistant DU145R80 xenograft model (Fig. 7b), where the endpoint size of control tumors was reached in 4 weeks, triple combination was able to completely block the tumor growth, compared with controls or other treated groups. VPA/SIM combination slightly reduced tumor growth only at last time point (day 32), while DTX alone clearly reduced growth in this tumor model. The combined treatment was well tolerated by both 22Rv1 and DU145R80 cells xenografted mice, as shown by the maintenance of body weight (Fig. 7a-b, *insets*) and by the absence of other signs of acute or delayed toxicity. Moreover, in 22Rv1 model, by calculating the percent change in tumor volume from the time of initial treatment (day 0) to the end of the study (day 21), VPA/SIM and triple combination treatment reduced the tumor burden by 15 and 29%, respectively, in spite of DTX that did not reduce tumor burden (Supplementary Fig. S10A). Conversely, in DU145R80 from day 7 to the end of the study (day 32) the triple combination reduced tumor burden by almost 90% compared with 40% reduction induced by VPA/SIM and about 62% by DTX (Supplementary Fig. S10B). In 22Rv1 xenograft model the synergistic intereaction between VPA/SIM and DTX was confirmed also by the evaluation of the TGD induced by the triple combination that reached a peak of more than 100% indicating that the mean rate of tumor growth in the control were approximately 2-fold higher, while compared to VPA/SIM the rate of control tumors was approximately 1.5-fold higher (Supplementary Fig. S10A). In DU145R80 xenograft model the mean rate of tumor growth in the control compared to the triple combination was approximately 3-fold higher (Supplemetary Fig. S9B). Moreover, we demonstrated also a clear increase of PARP cleavage in all the triple combination tumor samples compared with the other groups, in line with in vitro data showing increased apoptosis, paralleled by induction of DNA damage, measured as H2AX protein phosphorylation (Fig. 7c). Induction of AcH3 and HMGCR protein expression was used as a read-out of VPA and SIM, respectively (Fig. 7c) [24, 56]. Moreover, in agreement with our in vitro findings, a significant increase of phospho-YAP (Ser127) (Fig. 7c), together with a clear reduction of YAP-target genes BRC5 (Fig. 7d, left panel) and NANOg (Fig. 7d, right panel), were shown in both VPA/SIM and VPA/SIM/DTX treated mice compared with controls or DTX-treated tumors, further supporting our hypothesis. Furthermore, as shown in Fig. 7e VPA/SIM combination reduced cholesterol content also in vivo in tumor samples, and this effect is further potentiated in triple combination setting.

Next, we confirmed our previous observation also in 22Rv1 R_39 DTX resistant cells xenograft model. In details, fiftyfour mice were injected with 22Rv1 R_39 cells, and ten days after implantation the mice were randomly assigned to six groups to receive DTX, VPA and /or SIM, and triple combination, or their vehicles, for ten days, at the dosages indicated above for parental cells xenograft model. As shown in Fig. 7f VPA/SIM and triple combination produced a clear statistically significant tumor growth inhibition compared with control and single treatments groups. This effect was confirmed through the measurement of tumor volume (Fig. 7g, left panel) and tumor weight (Fig. 7g, right panel) ex vivo. The combined treatment was well tolerated by 22Rv1 R_39 cells xenografted mice, as shown by the maintenance of body weight (Fig. 7f, insets). Moreover, by calculating the percent change in tumor volume from the time of initial treatment (day 3) to the end of the study (day 13), VPA/SIM and triple combination treatment reduced the tumor burden by 29.2 and 34.2%, respectively, in spite of the other treated groups that did not reduce tumor burden (Supplemetary Fig.S10C). In addition, the mean rate of tumor growth in the control compared to the double and triple combination was approximately 1,3-fold higher for both groups (Supplemetary Fig. S10C). Interestingly, tumor incidence curves analyzing tumor engraftment (first appearance of a palpable mass) in cohorts of 9 mice/group injected with either 22Rv1 or 22Rv1 R_39 cells, showed that 22Rv1 cells xenograft model developed tumors with a latency of 8 days compared to 22Rv1 R_39 cells xenograft model, suggesting increasing aggressivity of the resistant subline, potentially related with CSC enrichment (Supplemetary Fig.S10D).

Overall we confirmed the efficacy of VPA/SIM combination to potentiate the antitumor effect of DTX and to revert DTX resistance also in vivo in PCa tumor models by targeting the MVP/YAP axis.

## Discussion

The success of most antitumor approaches, particularly in the metastatic setting, is judged on their ability to induce tumor shrinkage and/or prevent disease progression, thus improving survival. However, although eliminating the bulk of cancer cells, anticancer treatments generally select for resistant cell clones, leading to post-therapy relapse. CSCs enrichment has been associated to anticancer therapy resistance, and in PCa models several evidences suggest that CSCs contribute to resistance against chemotherapeutics, such as DTX or cabazitaxel, and androgen receptor inhibitors, such as enzalutamide [6, 57, 58].

In our study we report, for the first time, the synergistic antitumor interaction of two well-known generic drugs, used for years in clinical practice for medical indications other than cancer, such as the antiepileptic agent with HDACi activity VPA, and the cholesterol lowering agent SIM. In detail, we demonstrated the capacity of the combined approach to target the CSCs compartment in mCRPC models and unveiled a novel molecular mechanism underlying this synergism based on the inhibition of the oncogene YAP activity. Based on these evidences we then showed, both in vitro and in vivo models, the ability of VPA/SIM combination to sensitize PCa cells to a chemotherapeutic used in different treatment setting in this disease, such as DTX, and to revert DTX resistance.

Previous findings have demonstrated that stem-like populations persist in commercial PCa cell lines and are enriched by tumorsphere culture [59], indeed we demonstrated that VPA/SIM combination, compared to single agents, strongly inhibits CSC enriched first and second generation PCa spheroids formation, the latter even without repeated treatment, as well as stem cell frequency in limiting dilution assay, overall confirming the targeting of CSCs self-renewal capacity.

Mechanistically, we provided several evidences demonstrating that the VPA/SIM combined treatment induced increased YAP inhibitory phosphorylation, thus blocking its translocation into the nucleus impairing its transcriptional activity. Indeed, by overexpressing in PCa cells the constitutive active YAP5SA mutated form we reverted all these events, thus impairing VPA/SIM-induced effects on spheres formation and stem cell frequency, as well as on the potentiation of DTX antitumor activity and reversion of DTX-resistance.

YAP, and the highly related other transcriptional regulator TAZ (transcriptional coactivator with PDZ-binding motif) are the effectors of the Hippo pathway, controlling cell fate plasticity, polarity and organ size by shuttling between the cytoplasm and the nucleus, where they interact with TEAD (TEA domain) transcription factors family and others, regulating the transcription of genes involved in oncogenic features such proliferation, anti-apoptosis, cell mobility, and altered metabolism [46, 60]. Moreover, several evidences highlight the critical role of YAP in the generation of CSCs [61, 62], including in PCa models where YAP has been associated with cancer initiation and progression as well as with the onset of both castration and DTX resistance [30, 63, 64]. Consistently, Zhang et al. have shown a significant upregulation and hyperactivation of YAP in castration-resistant PCa compared to their levels in hormone-responsive PCa [65]. Similarly, in our study, by differential expression analysis from prostate adenocarcinoma TCGA database, we demonstrated that all the principal YAP transcriptional targets, CTGF, CYR61, BIRC5 and ANRDK1, were highly enriched in the patients with PCa tumors, compared with tumor free patients. In line with these data, our group, within a bioinformatics analysis on the activated-pathways related with CSCs generation and maintenance, has previously identified the Hippo pathway as strongly altered and associated with bad prognosis in patients with PCa [9].

Here we also demonstrated that the synergistic inhibition of MVP by the combined treatment is the critical upstream event leading to YAP impairment. Indeed, we demonstrated that all the molecular events and the antitumor effects induced by VPA/SIM combination alone, or plus DTX, reported above, were antagonized by the mevalonic acid, that bypasses the inhibition of HMGCR, the first rate limiting enzyme of MVP and the target of statins. Similarly, bypassing downstream in MVP the prenylation arm inhibition, by using GGOH, also reverts the VPA/SIM-induced inhibition of YAP activation.

MVP, being the metabolic route for the production of steroid-based hormones, is directly connected with PCa initiation and progression, and has been associated with CSCs generation in several tumor types [12, 66, 67]. Notably, many studies imply that Hippo is one of the main pathways influenced by a functionally active MVP via the prenylation of the Rho GTPase. In detail, MVP promotes nuclear localization and activation of YAP/TAZ independently of the canonical LATS1/2 kinase regulation [46], thus controlling CSCs fate [61]. By using statins as well as inhibitors of the prenylation arm such as ZOL, all these effects are reverted [61].

Accumulating evidences indicate that in addition to the “traditional” regulatory schemes, cholesterol homeostasis is also under the control of epigenetic mechanisms such as histone acetylation [68]. On the other hand, epigenetic consequences from inhibitors of MVP have been also recently showed [69]. Indeed, several studies reported the antitumor efficacy of combining HDACi, including VPA, with statins, or other MVP inhibitors, in different cancer models, including PCa [31, 70–74]. For example, we previously demonstrated in vitro and in vivo models of mCRPC, the synergistic antitumor effect between ZOL and the HDACi panobinostat [31]. Here we also report for the first time the ability of an HDACi [39, 75], such as VPA, to completely revert the resistance to SIM in the PCa DU145R80-resistant model. Moreover, dual-targeting HDAC/HMGCR inhibitors have been synthetized and successfully tested as anti-tumor agents [71]. Furthermore, a large population-based study strongly suggests a lowered risk for PCa among users of drugs with HDAC inhibitory activity, with a slight reduction of the overall PCa risk for men stratified by the concomitant use of statins [76]. The critical role of prenylation arm inhibition has been suggested as potential mechanism of the observed synergism between HDACi and MVP inhibitors in several reports [70, 73]. However, in the present study we were the first, to our knowledge, to report a specific molecular interaction of VPA and SIM, converging on the inhibition of YAP activation and leading to CSCs population impairment. Interestingly, very recent observations suggested that epigenetic drugs such as bromodomain and extraterminal (BET) inhibitors, opposing the effect of bromodomanin containing protein (BRD) that are transcription regulators binding acetylated histones, or HDACi, are effective in targeting YAP activation in cancer cells addicted to this pathway [77].

Mechanistically, guided by the IPA network obtained by combining “HDAC inhibitors” and “MVP enzymes” search, we focused on the energy sensor kinase AMPK, that, by inducing a direct inhibitory phosphorylation of HMGCR, is a well-known upstream regulator of MVP. We demonstrated that VPA/SIM synergistically induced the phosphorylation and activation of AMPK, in line with previous reports also showing the ability of HDACi and statins, as single agents and in combination, to activate AMPK, a mechanism reported to contribute to antitumor effect via-autophagy [43, 44, 74]. However, we suggested a different mechanism, highlighting, for the first time, that VPA/SIM-induced AMPK activation is paralleled by the increased inhibitory phosphorylation of HMGCR and YAP. In this regard, are of particular interest the evidences demonstrating the ability of AMPK to regulate the Hippo pathway by directly inducing YAP inhibitory phosphorylation, thus being involved in CSCs regulation [47, 49]. Indeed, we also showed that pharmacological inhibition of AMPK partially reverts VPA/SIM synergistic inhibition of cell proliferation and apoptosis in PCa cells in both adherent condition and 3D spheroids. Notably, by interrogating again IPA combining “AMPK” and “HMGCR” search, we reveal an additional network including HDAC2, the target of VPA, all MVP enzymes, and the three YAP targets CYR61, CTGF and BIRC2.

**Fig. 8:**
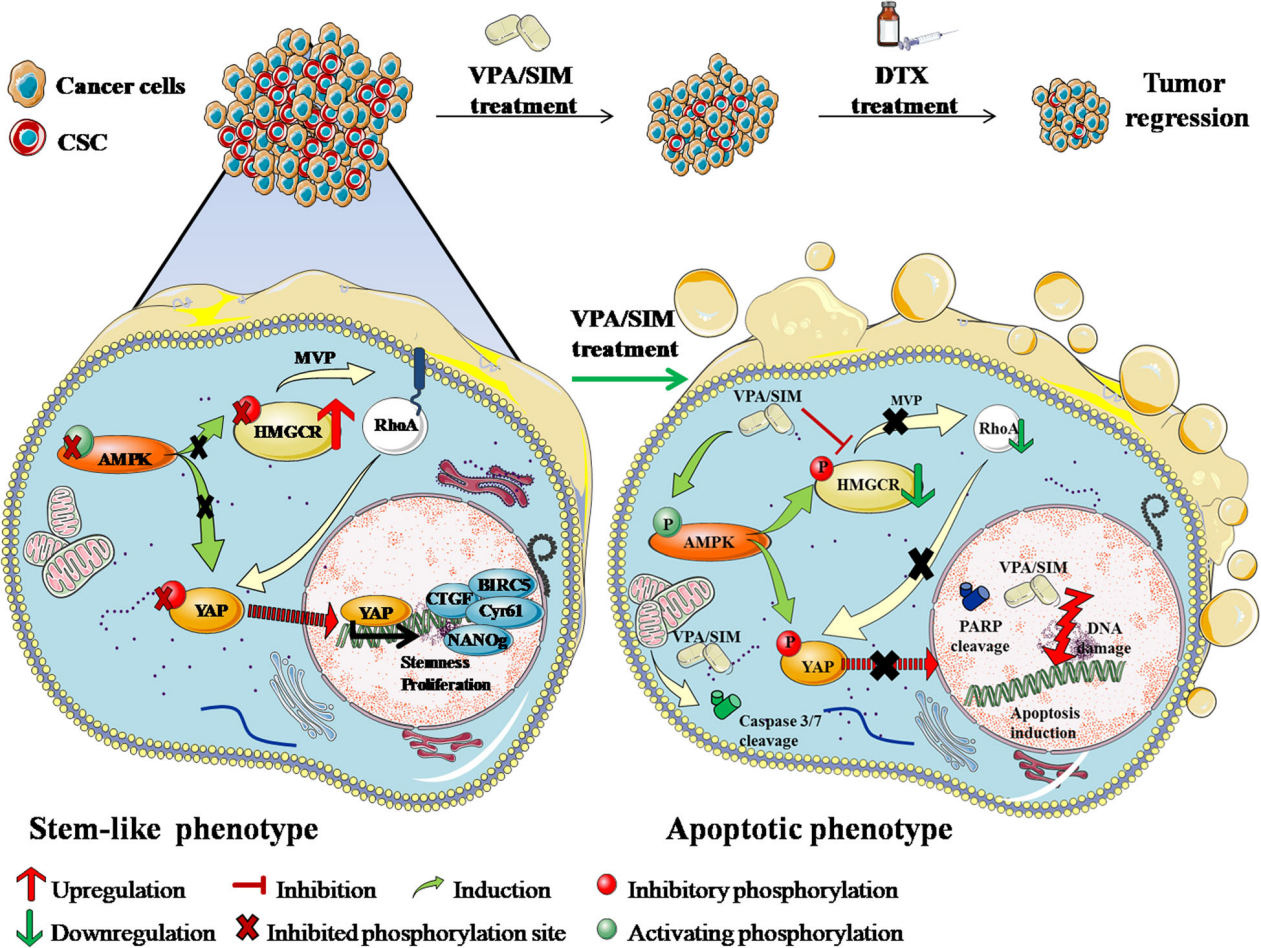
Graphical model describing the mechanism by which valproic acid/simvastatin combination by inhibiting the mevalonate pathway/YAP axis, specifically targets cancer stem cells, thus potentiating docetaxel antitumor effect

In summary, our hypothesis is that the dysregulation of the inhibitory activity of AMPK and/or hyperactivation of MVP, leading to YAP activation, contribute to the onset and maintenance of CSC, and that VPA/SIM, by regulating these pathways, specifically target CSCs population, thus potentiating DTX and reversing DTX resistance (Fig. 8).

We presented several evidences demonstrating indeed that VPA/SIM potentiated the antitumor effect of DTX both in vitro, in several CRPC models, and in two in vivo xenograft models and that this effect is dependent on the targeting of CSCs compartment via YAP-inhibition. Moreover, we also showed that VPA/SIM combination is able to revert DTX-resistance both in vitro and in in vivo in an additional xenograft model, again by targeting YAP hyperactivation.

DTX remains a current standard therapeutic option for mCRPC, however it only increases overall survival by on average 2.5 months, and those patients who initially respond eventually develop resistance [78]. Moreover, the onset of DTX-resistance and CRPC progression are driven by complex genetic and epigenetic mechanisms that remain poorly understood [79].

In our study we added new insight in the mechanism of DTX-resistance and also suggest a potential novel combinatory approach in mCRPC patients that warrant further clinical evaluation. Notably, the synergistic anti-tumor effect of VPA/SIM combination occur using low doses of both agents, easily reached in the plasma of patients treated for epilepsy or for high-cholesterol, respectively [32, 54]. Moreover, the synergistic interaction is not dependent on the treatment schedule used, an observation that could be clinically relevant because a less stringent condition of drug administration would make this combination easily adaptable for clinical application. Furthermore, we did not observe any pro-apoptotic effect in normal epithelial prostate cells, suggesting a good therapeutic index for this combination.

Several ongoing clinical trials are investigating HDACi in PCa patients, although their clinical efficacy in monotherapy, as also shown in other solid tumors, is limited, and an early clinical study in combination with DTX has recently been concluded [80]. We are currently evaluating the potential of VPA, at anti-epileptic dosage, in three ongoing clinical trials, demonstrating feasibility and safety of this agent in different clinical setting and in combination with different antitumor approaches [81, 82] [Revolution, EudraCT Number: 2018–001414-15].

Overall, the combination of two generic drugs such as VPA and SIM, can be easily translated in an early clinical trial since both agents are orally available and are drugs used chronically and safely for a long time and in a large number of people. Furthermore, on the bases of our data, the selection or stratification of mCRPC patients on the basis of YAP activation (ie. overexpression, nuclear localization, phosphorylation, expression of target genes) could be proposed.

Anyhow, because CSCs population and YAP activation have been associated to mechanisms of resistance against several anticancer agents, we suggest that DTX potentiation and DTX-resistance reversion by VPA/SIM can be considered a proof of concept to be extended to other antitumor approach. We are indeed currently testing this combination in other cancer models and in combination with other anticancer drugs.

## Supplementary information

**Additional file 1.** Supplementary Methods.

**Additional file 2: Supplementary Table S1.** Screening of PCa cell lines, antiproliferative effect of drugs alone. **Supplementary Table S2.** Antiproliferative effect induced by VPA in combination with SIM on PCa cell lines. **Supplementary Table S3.** Antiproliferative effect induced by by VPA in combination with SIM accordingly to different schedules of exposure in PC3 and 22Rv1 cell lines.

**Additional file 3: Supplementary Figure S1.** Soft agar clonogenic assay of DU145 (A) and DU145 R80 cells (B), untreated or treated with VPA and SIM alone and in combination at 1 mM and 0.625 μM respectively (IC_25_^96h^ values) for both cell lines. Colonies of > 100 μm were scored by a colony counter. Right: images from a representative experiment; left: values are the mean ± S.D. from at least three independent experiments. **Supplementary Figure 2.** A. Characterization of the indicated prostate cancer cells for their ability to growth in low attach condition ad 3D-spheroids; all cell lines (40,000 cell/ml) were plated in low attach support and sphere medium for 72 h. B. Nanog (left panel) and OCT4(right panel) mRNA expression evaluated by RT-PCR at basal level in 22Rv1 in cell adhesion condition, in 1st generation spheres and in 2nd generation spheres. β-actin was used as housekeeping control gene to normalize RT-PCR reactions. C. Basal expression of NANOg evaluated by western blotting in 22Rv1 cells in cell adhesion condition, in 1st generation spheres and in 2nd generation spheres. Γtubulin was used as loading control. D. Surface marker expression (CD44 and CD113) was determined by flow cytometry on 22Rv1cells at basal level in 22Rv1 in both cell adhesion condition and 1st generation spheres. E. CTGF mRNA expression evaluated by RT-PCR at basal level in 22Rv1 in both cell adhesion condition and in 1st generation spheres. β-actin was used as housekeeping control gene to normalize RT-PCR reactions Statistically significant results are reported (*** indicates *P* < 0.0005, ** indicates *P* < 0.005 and * indicates *P* < 0.05). **Supplementary Figure S3.** A. Expression of acetyl histone H3 (AcH3) evaluated by western blot in the indicated cell lines, untreated or treated with VPA and SIM alone or in combination at the IC5096h for 24 h,; ponceau red serves as control for equal protein loading. B. HMGCR mRNA expression evaluated by RT-PCR in 22Rv1 cells untreated or treated for the indicated time points with VPA or SIM at the IC5096h; the values represent the means±S.D. of technical triplicates. C. HDAC1 and HDAC2 mRNA expression evaluated by RT-PCR in 22Rv1 cells untreated or treated 4 h with VPA, SIM, or the combination, at the IC_50_^96h^; the values represent the means ± S.D. of technical triplicates. Statistically significant results are reported (*** indicates *P* < 0.0005, ** indicates *P* < 0.005). D. RhoA subcellular localization assessed by western blotting, in 22Rv1 cells treated with VPA (left panel) or SIM (right panel) at I IC_50_^96h^ in the absence or presence of either Mev (100 μM) or GGOH (20 μM 1 h before either treatment); the cytosolic and membrane fractions are denoted by C and M, respectively; cytosolic marker: GAPDH; membrane marker: VDAC. **Supplementary Figure S4.** VPA/SIM treatment regulates YAP phosphorilation acting through AMPK/MVP axis.A. Expression of phospho-AMPK (p-AMPK) and phospho-HMGCR (pHMGCR) evaluated by western blotting, in 22Rv1 cells untreated or treated for the indicated time points with VPA or SIM at the IC_50_^96h^; CDK4 serves as control for equal protein loading. B. Western blotting analysis of p53, phospho-AMPK (pAMPK), phopsho ACC (pACC) phospho-HMGCR (pHMGCR), phospho-YAP (pYAP) and YAP in 22Rv1 treated with VPA and/or SIM at the IC5096h doses for 24 h. Extracts (30 μg) were resolved by SDS-PAGE and immunoblotted using specific antibodies, βactin or γtubulin were used as loading control. C. Expression of p53, phospho-AMPK (pAMPK), phospho-HMGCR (pHMGCR), phospho-YAP (pYAP) and YAP evaluated by wetern blotting in PC3 cells untreated or treated for 4 h with VPA, SIM or the combination at IC5096h doses for 4 h. Basal LNCaP extract was used as Positive Control (PC) of p53 expression. Extracts (30 μg) were resolved by SDS-PAGE and immunoblotted using specific antibodies. Βactin was used as loading control. D. Western blotting analysis of pYAP and YAP in PC3 cells treated with VPA and/or SIM at the IC5096h doses for 24 h. **Supplementary Figure S5**. VPA/SIM treatment regulates YAP subcellular localization acting through AMPK/MVP axis. A. Western blotting analysis of cytoplasmatic YAP in 22Rv1 cells treated with VPA and/or SIM at the IC_50_^96h^ doses for 24 h ± mevalonic acid (Mev) (100 μM) and ± GGOH (20 μM 1 h before either treatment). γTubulin was used as loading control. B. Western blotting analysis of nuclear YAP in 22Rv1 cells treated with VPA and/or SIM at the IC_50_^96h^ doses for 24 h, ± Mev (100 μM). PARP was used as loading control. C. Visual representation of the network generated by Ingenuity Pathway Analysis (IPA) combining “MVP enzymes” and “AMPK” search, which includes in bold YAP target genes (CTGF, CYR61 and BIRC5), HDAC2, MVP regulating genes (Srbp, SREBF1 and SREBF2) and AMPK related genes (i.e ACAC); network genes are visualized by proper symbols, which specify the functional nature of the correspondent protein; each node represents a gene and its direct (represented by solid lines) and indirect (represented by dotted lines) association with other genes. D. AMPK, HMGCR and YAP protein basal expression evaluated by western blotting in PC3, 22Rv1, DU145, DU145R80 and LNCaP prostate cancer cells and in EPN normal epithelial prostate cells; GAPDH was used as the protein loading control. E. Expression of p53, p-AMPK and phospho-YAP (pYAP) evaluated by wetern blotting in LNCaP cells untreated or treated for 4 h with VPA, SIM or the combination at IC_50_^96h^ doses. βACTIN was used as control for equal protein loading. **Supplementary Figure S6.**. Pharmacological inactivation of AMPK with compound C reverts the antiproliferative and apoptotic effect induced by VPA/SIM combination. **A**. 22Rv1 cells untreated or treated for 72 h with VPA and/or SIM at the IC5096 h doses ± compound C (CC) (10 μM). Cell growth expressed as percentage of control was assessed by sulforhodamine B colorimetric assay. The values, expressed as percentage of control, are the means ± S.D. from at least three independent experiments. **B**. Apoptosis was evaluated by Caspase 3/7 activity assay in 22Rv1 cells untreated or treated for 24 h with VPA and/or SIM at the IC5096 h doses ± CC 0,5 μM (left panel) and 1 μM (right panel). **C**. 22Rv1 cells were seeded to form spheres D: cell seeded to form 1st generation speheres and concomitantly untreated or treated with VPA and /or SIM at the IC5096 h doses ± CC (1 μM) for 72 h, then disaggregated and plated again to form 2nd generation spheres without additional treatment for 72 h. Spheroids viability was assessed by luminescence assay. The values are the means ± S.D. from at least three independent experiments. Statistically significant results are reported (*** indicates *P* < 0.0005, ** indicates *P* < 0.005 and * indicates *P* < 0.05). **Supplementary Figure S7.** Prostate adenocarcinoma Cancer Genome Atlas (TCGA): expression of YAP-target genes CTGF, CYR61, BIRC5 and ANKRD, in in live and dead patients, performed by R2 platform of analysis. **Supplementary Figure S8.** VPA/SIM combination synergistically interacts with DTX on prostate cancer 2D and 3D models. **A**. Apoptotsis evaluated by flow cytometry analysis in DU145, PC3 and EPN cells were treated or untreated for 48 or 72 h with VPA, SIM, DTX, dual VPA/SIM or triple combinations at IC_50_^96h^, expressed as % of hypodiploid population (sub-G0-G1). **B**. Apoptosis evaluated by Caspase 3/7 luminescence activity assay in DU145, PC3 and EPN cells, untreated or treated for 24 h with VPA, SIM, DTX dual VPA/SIM or triple combinations at IC_50_^96h^. C. 22Rv1 microtissues generated in 72 h by GravityPlus hanging drop system in the absence or the presence of VPA, SIM, DTX, dual VPA/SIM or triple combinations, then transferred into GravityTrap plates where cell viability (bars) was evaluated by luminescence assay. D. 22Rv1 microtissues generated in 72 h as above, in the absence of drugs, were the transferred into GravityTrap and treated with VPA, SIM, DTX, dual VPA/SIM or triple combinations at IC_50_^96h^ doses, after additional 72 h cell viability (bars) was evaluated by luminescence assay; the values represent means ± S.D. of technical triplicates. E. 22Rv1 cells (40,000/mL) were seeded in sphere medium in low attachment 96 multiwell and left untreated or treated with VPA, SIM, DTX, dual VPA/SIM or triple combinations at IC5096h as follows: Spheres A: cells plated and concomitantly treated for 72 h; Spheres B: cells grown for 72 h then disaggregated and plated again in the absence or presence of drugs for additional 72 h; Spheres C: spheres grown for 72 h and then left untreated or treated for additional 72 h; viability was assessed by luminescence assay; values are the means±S.D. from at least three independent experiments. Statistically significant results are reported (*** indicates *P* < 0.0005, ** indicates *P* < 0.005 and * indicates *P* < 0.05). Lower panel: images of double and triple combinations effect from a representative experiment. F. Tumor growth delay (TGD), determined, in 22Rv1 cells as %TGD = [(T − C) /C] × 100, where T and C are the mean times expressed in days for the treated or control groups, respectively, to reach a defined tumor volume (see Materials and Methods). Statistically significant results are reported (*** indicates *P* < 0.0005, ** indicates *P* < 0.005 and * indicates *P* < 0.05). **Supplementary Figure S9**. **A.** Surface marker expression (CD44 and CD113) was determined by flow cytometry on 22Rv1cells at basal level in 22Rv1 R_39 in both cell adhesion condition and 1st generation spheres. **B.** Images from the experiment in Fig. 6f, of 22Rv1_wt, 22Rv1 DTX_r39 and YAP5SA –transfected 22Rv1 DTX_r39 SphereD. 40,000/mL cells were seeded ins phere medium in low attachment 96 multiwell untreated or treated with VPA, SIM and DTX at the respective IC5096h, then disaggregated and plated again for 72 h without additional treatment. **Supplementary Figure S10. A.** Percent change in tumor volume average (left panel) of 22Rv1 xenografts from the time of initial treatment (day 0) to the end of the study (day 21) for each treatment group compared to vehicles group; tumor growth delay (TGD) (right panel), determined in 22RV1 cells as %TGD = [(T − C) /C] × 100, where T and C are the mean times expressed in days for the treated or control groups, respectively, to reach a defined tumour volume (see Methods); representative images of tumors from each treatment group collected at the end of the treatment. **B**. Percent change in tumor volume average (left panel) from each group of DU145R80 model at day 7 and day 32 were compared and presented as percentages of vehicle; tumor growth delay (TGD) (right panel), determined in DU145R80 cells as %TGD = [(T − C) /C] × 100, where T and C are the mean times expressed in days for the treated or control groups, respectively, to reach a defined tumor volume (see Methods); representative images of tumors from each treatment group collected at the end of the treatment. **C.** Percent change in tumor volume average (left panel) of 22Rv1 R_39 xenografts from the time of initial treatment (day 3) to the end of the study (day 13) for each treatment group compared to vehicles group; tumor growth delay (TGD) (right panel), determined in 22RV1 R_39 cells as %TGD = [(T − C) /C] × 100, where T and C are the mean times expressed in days for the treated or control groups, respectively, to reach a defined tumor volume (see Materials and Methods); representative images of tumors from each treatment group collected at the end of the treatment. **D.** Incidence curves analyzing tumor engraftment (first appearance of a palpable mass) in cohorts of 9 mice/group injected with 22Rv1 or 22Rv1 R_39 cells. As assessed by Log Rank test, the difference between the curves were highly significant (*P* < 0.0237).

## Data Availability

Most of the data generated or analysed during the present study are included in this published article. All raw data produced by Calcusyn software on the interaction between VPA, SIM and DTX evaluated in vitro as well as NMR row data will be available in publicly accessible resources.

## Abbreviations

PCa: Prostate cancer
mCRPC: Castration-resistant metastatic disease
DTX: Docetaxel
CSCs: Cancer stem cells
MVP: Mevalonate pathway
HMGCR: 3-hydroxy-3-methylglutaryl-coenzyme A reductase
HDACi: Histone deacetylase inhibitors
VPA: Valproic acid
SIM: Simvastatin
YAP: Yes-associated protein
CIs: Combination indexes
DRI: Dose reduction index
PBS: Phosphate Buffer saline
BSA: Bovine serum albumine
NMR: Nuclear magnetic resonance spectroscopy
PI: Propidium iodure
MTT: 3-(4,5-dimethylthiazol-2-yl)-2,5-diphenyltetrazolium bromide
RI: Resistance index
SDS: Sodium Dodecyl Sulphate
PAGE: PolyAcrylamide Gel Electrophoresis
FGF: Fibroblast growth factor
EGF: Epidermal growth factor
RT: Room temperature
DAPI: 4′,6-diamidin-2-fenilindolo
DMSO: Dimethyl sulfoxide
DMEM: Dulbecco’s Modified Eagle Medium
TV: Tumor volume
TGD: Tumor growth delay
ZOL: Zoledronic acid
Mev: Mevalonic acid
GGOH: Geranylgeranyol
HDAC1: Histone deacetylase1
HDAC2: Histone deacetylase2
HDAC3: Histone deacetylase3
HDAC6: Histone deacetylase6
HMG-CoA synthase: Hydroxymethylglutaryl-CoA synthase
SREBF: Sterol regulatory element-binding protein 1
AMPK: AMP-activate protein kinase
CTGF: Connective tissue growth factor
Cyr61: Cysteine-rich, angiogenic inducer, 61
BIRC5: Baculoviral IAP repeat containing 5
ACC: Acetyl-CoA carboxylase
ANRDK1: Ankyrin repeat domain containing protein 1
TCGA: The Cancer Genome Atlas
γH2AX: Phosphorylated histone H2AX
PARP: Poly [ADP-ribose] polymerase 1
AcH3: Acetylated Histone 3
NANOg: Nanog homeobox
OCT4: Organic cation/carnitine transporter4
TAZ: Transcriptional coactivator with PDZ-binding motif
TEAD: TEA domain
LATS1/2: Large tumor suppressor kinase ½
BET: Bromodomain and extraterminal
