# Supplementary material for: Synergistic antitumor interaction of valproic acid and simvastatin sensitizes prostate cancer to docetaxel by targeting CSCs compartment via YAP inhibition
Source: J Exp Clin Cancer Res. 2020 Oct 8;39:213. doi: 10.1186/s13046-020-01723-7 (PMC7545949; doi:10.1186/s13046-020-01723-7)
Supplement: Supplementary file 1 — Additional file 1. Supplementary Methods. [file 13046_2020_1723_MOESM1_ESM.docx]

**Supplementary Methods**

**Reagents**

Valproic acid (VPA) was purchased from Enzo Life Sciences (Farmingdale, NY, USA), Simvastatin (#1693) was purchased from Biovision Incorporated (Milpitas, CA, USA) and Docetaxel (#11637) was purchased from Cayman Chemical Company (Ann Arbor, MI, USA) Mevalonic Acid (Mev) (#41288) and Geranylgeraniol (GGOH) (#G3278) were purchased from Sigma- Aldrich (St. Louis, MO, USA).

**List of antibody and probes**

The primary antibodies used were the following: p53 (#9282), AcH3 (#9677), PARP (#9542), phospho-AMPK (Thr172) (#2531), AMPKα (#2532), phospho-ACC (Ser79) (#3661), phospho-YAP (Ser127) (#4911), YAP (#4912), , and γ-Tubulin (#5886) were purchased from Cell signaling Technology (Danvers, MA, USA). Phospho HMGCR (Ser872) (ab215437), HMGCR (ab214018), GAPDH (ab8245), γH2Ax (phospho S139) (ab11174) and β-actin (Ab8227), were purchased from Abcam (Cambridge, UK). RhoA (sc-418) and CDK-4 ( sc23896) were purchased from Santa Cruz Biotechnology (Dallas, TX, USA). Antibodies used for flow cytometry experiments: PE conjugated Anti-human CD133 (Miltenyi Biotec S.r.l., Bergisch Gladbach, North Rhine-Westphalia, Germany) and FITC conjugated Anti-human CD44 (BD Pharmingen, Franklin Lakes, NJ, USA).

Probes used were the following: HMGCR (QT00004081), CTGF (QT00052899), Cyr61 (QT00003451), BIRC5 (QT00081186), NANOg (QT01025850), OCT4 (Hs04260367_gH), HDAC1 (Hs02621185_s1), HDAC2 (Hs00231032_m1), purchased from Qiagen (Valencia, CA, USA).

**Cell culture conditions**

PC3, DU145, LNCaP and 22Rv1 cell lines were purchased from American Type Culture Collections (ATCC) and authenticated. The cells have been authenticated with short tandem repeat profile generated by LGC Standards (Teddington, UK). EPN cells were kindly provided by Dr. D Tramontano, University of Naples Federico II, Naples, Italy [*Sinisi AA et al.* [*In Vitro Cell Dev Biol Anim.*](https://www.ncbi.nlm.nih.gov/pubmed/12026165)*2002*]. ZOL-resistant DU145R80 cells were obtained as previously described [*Milone et al . Cell Death Dis. 2013*] and authenticated. All the cells lines were grown in RPMI (Roswell Park Memorial Institute) supplemented with 10% fetal bovine serum (FBS, Cambrex, Belgium) heat-inactivated, 50 units/ml penicillin (Cambrex, Belgium), 500 g/ml streptomycin (Cambrex, Belgium), and glutamine 4 mM. The cells were grown in a humidified atmosphere composed of 95% air and 5% CO2 at 37°C.

**Microtissue formation assay**

Prostate cancer cell lines 22RV-1 and DU145-R80 were cultured as microtissues by the GravityPLUS™ Hanging Drop System (InSphero AG, Wagistrasse, Switzerland). Cells were plated following instructions from InSphero kit and untreated or treated with drugs, as described above. Within 2-4 days, a single microtissue forms in each drop. Once the microtissues are formed they can easily be harvested into the GravityTRAP™ plate with a simple media addition step. 3D microtissues were maintained in the incubator and scored by CellTiter-Glo® 3D Cell Viability Assay (Promega, Madison, WI, USA) at 96h by using a Multilabel Reader VICTOR X4 2030 (PerkinElmer, Waltham, MA, USA).
