# Supplementary material for: Synergistic antitumor interaction of valproic acid and simvastatin sensitizes prostate cancer to docetaxel by targeting CSCs compartment via YAP inhibition
Source: J Exp Clin Cancer Res. 2020 Oct 8;39:213. doi: 10.1186/s13046-020-01723-7 (PMC7545949; doi:10.1186/s13046-020-01723-7)
Supplement: Supplementary file 2 — Additional file 2: Supplementary Table S1. Screening of PCa cell lines, antiproliferative effect of drugs alone. Supplementary Table S2. Antiproliferative effect induced by VPA in combination with SIM on PCa cell lines. Supplementary Table S3. Antiproliferative effect induced by by VPA in combination with SIM accordingly to different schedules of exposure in PC3 and 22Rv1 cell lines. [file 13046_2020_1723_MOESM2_ESM.ppt]

## Slide 1
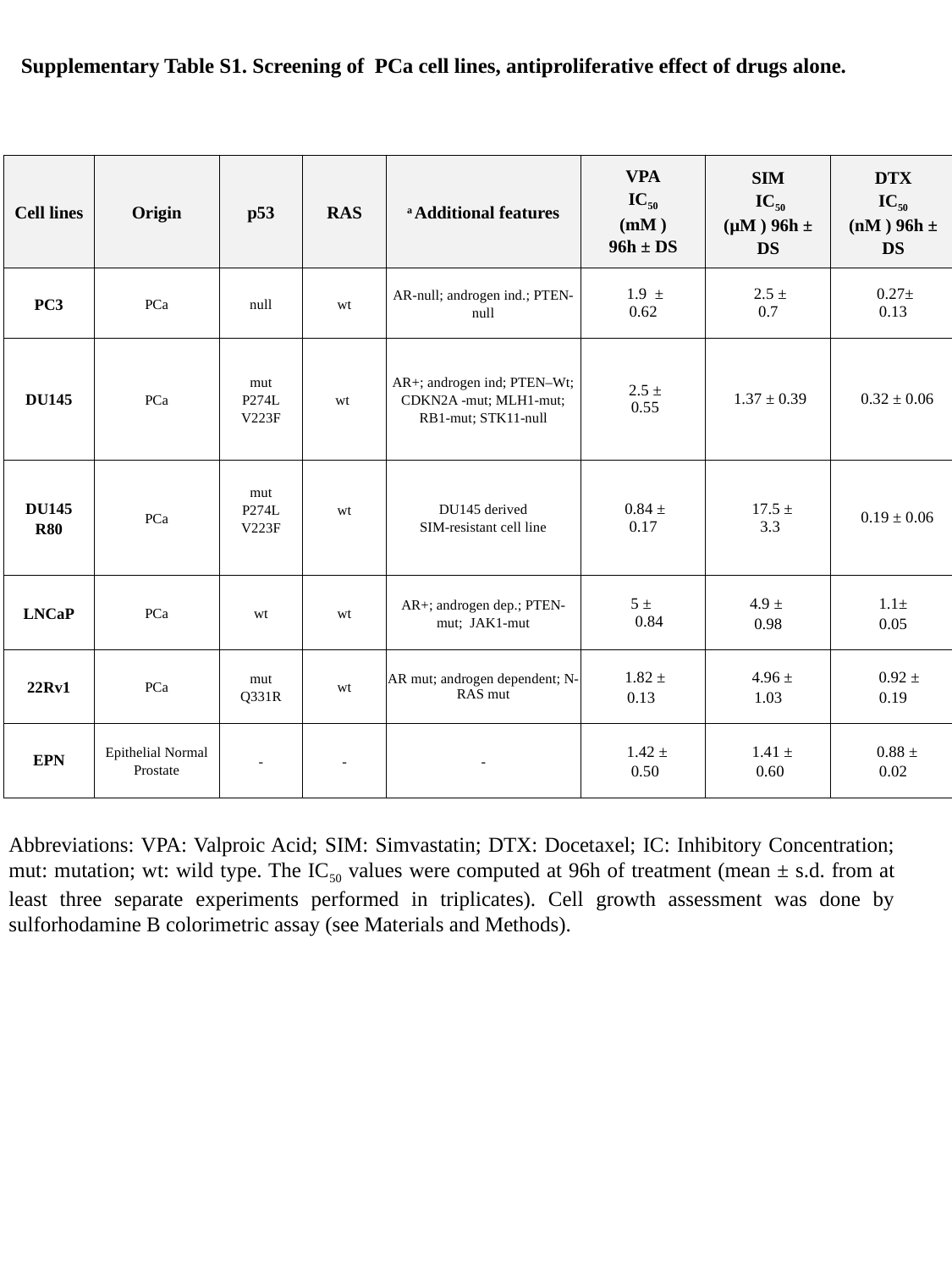

Supplementary Table S1. Screening of PCa cell lines, antiproliferative effect of drugs alone.
| Cell lines | Origin | p53 | RAS | a Additional features | VPA IC50 (mM ) 96h ± DS | SIM IC50 (µM ) 96h ± DS | DTX IC50 (nM ) 96h ± DS |
| --- | --- | --- | --- | --- | --- | --- | --- |
| PC3 | PCa | null | wt | AR-null; androgen ind.; PTEN-null | 1.9 ± 0.62 | 2.5 ± 0.7 | 0.27± 0.13 |
| DU145 | PCa | mut P274L V223F | wt | AR+; androgen ind; PTEN–Wt; CDKN2A -mut; MLH1-mut; RB1-mut; STK11-null | 2.5 ± 0.55 | 1.37 ± 0.39 | 0.32 ± 0.06 |
| DU145 R80 | PCa | mut P274L V223F | wt | DU145 derived SIM-resistant cell line | 0.84 ± 0.17 | 17.5 ± 3.3 | 0.19 ± 0.06 |
| LNCaP | PCa | wt | wt | AR+; androgen dep.; PTEN-mut; JAK1-mut | 5 ± 0.84 | 4.9 ± 0.98 | 1.1± 0.05 |
| 22Rv1 | PCa | mut Q331R | wt | AR mut; androgen dependent; N-RAS mut | 1.82 ± 0.13 | 4.96 ± 1.03 | 0.92 ± 0.19 |
| EPN | Epithelial Normal Prostate | - | - | - | 1.42 ± 0.50 | 1.41 ± 0.60 | 0.88 ± 0.02 |
a For PC3, DU145, LNCaP and 22RV1 information has been derived from www.sanger.ac.uk.
Abbreviations: VPA: Valproic Acid; SIM: Simvastatin; DTX: Docetaxel; IC: Inhibitory Concentration; mut: mutation; wt: wild type. The IC50 values were computed at 96h of treatment (mean ± s.d. from at least three separate experiments performed in triplicates). Cell growth assessment was done by sulforhodamine B colorimetric assay (see Materials and Methods).

## Slide 2
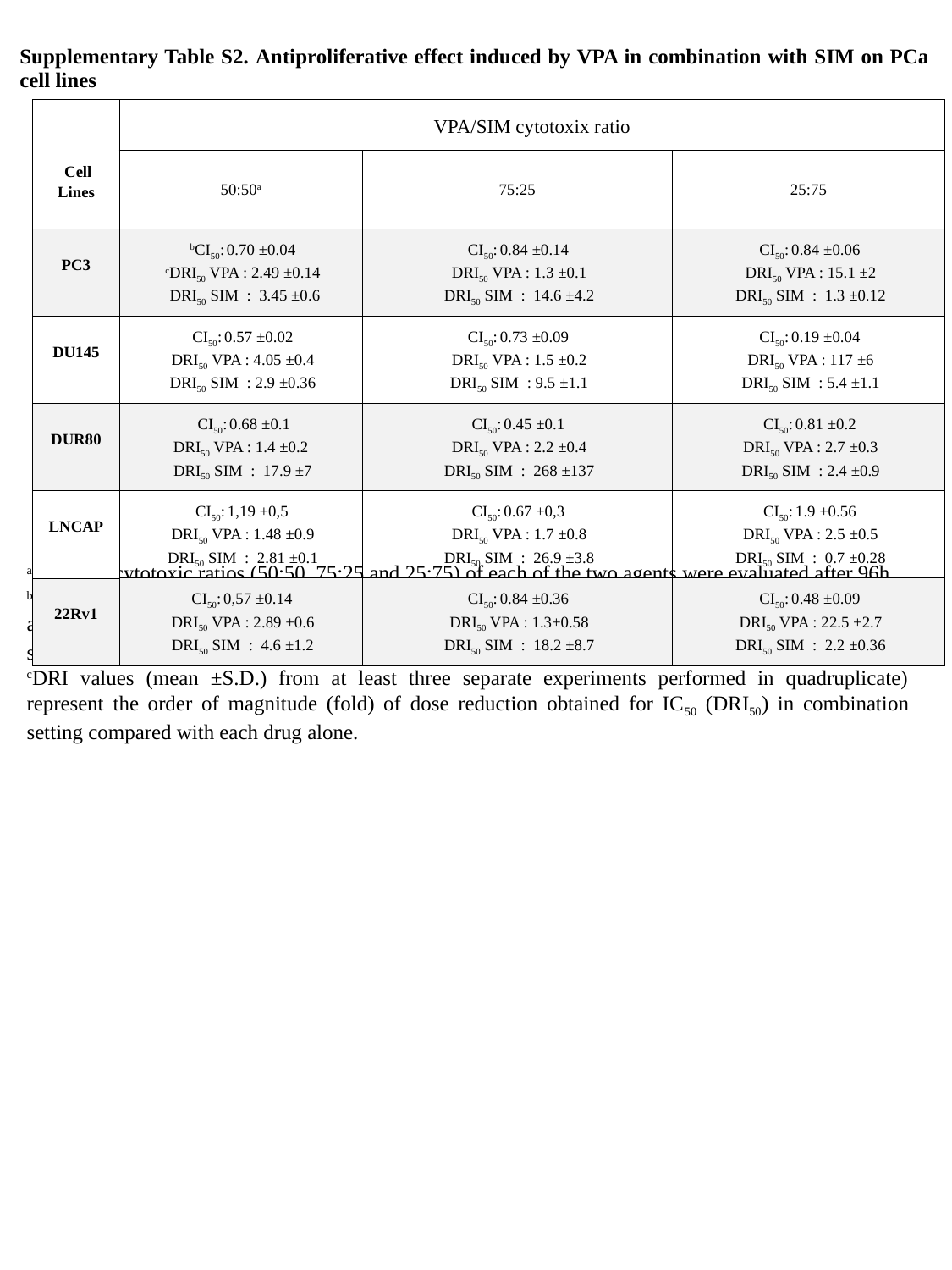

Supplementary Table S2. Antiproliferative effect induced by VPA in combination with SIM on PCa cell lines
| | VPA/SIM cytotoxix ratio | | |
| --- | --- | --- | --- |
| Cell Lines | 50:50a | 75:25 | 25:75 |
| PC3 | bCI50: 0.70 ±0.04 cDRI50 VPA : 2.49 ±0.14 DRI50 SIM : 3.45 ±0.6 | CI50: 0.84 ±0.14 DRI50 VPA : 1.3 ±0.1 DRI50 SIM : 14.6 ±4.2 | CI50: 0.84 ±0.06 DRI50 VPA : 15.1 ±2 DRI50 SIM : 1.3 ±0.12 |
| DU145 | CI50: 0.57 ±0.02 DRI50 VPA : 4.05 ±0.4 DRI50 SIM : 2.9 ±0.36 | CI50: 0.73 ±0.09 DRI50 VPA : 1.5 ±0.2 DRI50 SIM : 9.5 ±1.1 | CI50: 0.19 ±0.04 DRI50 VPA : 117 ±6 DRI50 SIM : 5.4 ±1.1 |
| DUR80 | CI50: 0.68 ±0.1 DRI50 VPA : 1.4 ±0.2 DRI50 SIM : 17.9 ±7 | CI50: 0.45 ±0.1 DRI50 VPA : 2.2 ±0.4 DRI50 SIM : 268 ±137 | CI50: 0.81 ±0.2 DRI50 VPA : 2.7 ±0.3 DRI50 SIM : 2.4 ±0.9 |
| LNCAP | CI50: 1,19 ±0,5 DRI50 VPA : 1.48 ±0.9 DRI50 SIM : 2.81 ±0.1 | CI50: 0.67 ±0,3 DRI50 VPA : 1.7 ±0.8 DRI50 SIM : 26.9 ±3.8 | CI50: 1.9 ±0.56 DRI50 VPA : 2.5 ±0.5 DRI50 SIM : 0.7 ±0.28 |
| 22Rv1 | CI50: 0,57 ±0.14 DRI50 VPA : 2.89 ±0.6 DRI50 SIM : 4.6 ±1.2 | CI50: 0.84 ±0.36 DRI50 VPA : 1.3±0.58 DRI50 SIM : 18.2 ±8.7 | CI50: 0.48 ±0.09 DRI50 VPA : 22.5 ±2.7 DRI50 SIM : 2.2 ±0.36 |
aDifferent cytotoxic ratios (50:50, 75:25 and 25:75) of each of the two agents were evaluated after 96h.
bCI values (mean ±S.D.) from at least three separate experiments performed in quadruplicate) computed at 50% of cell kill (CI50) by CalcuSyn software (Biosoft,Cam- bridge, UK); CIs values <0.8 indicate strong synergism; CIs <0.9 sinergysm; CIs between 0.9 and 1.2 additivity; CI> 1.2 antagonism;
cDRI values (mean ±S.D.) from at least three separate experiments performed in quadruplicate) represent the order of magnitude (fold) of dose reduction obtained for IC50 (DRI50) in combination setting compared with each drug alone.

## Slide 3
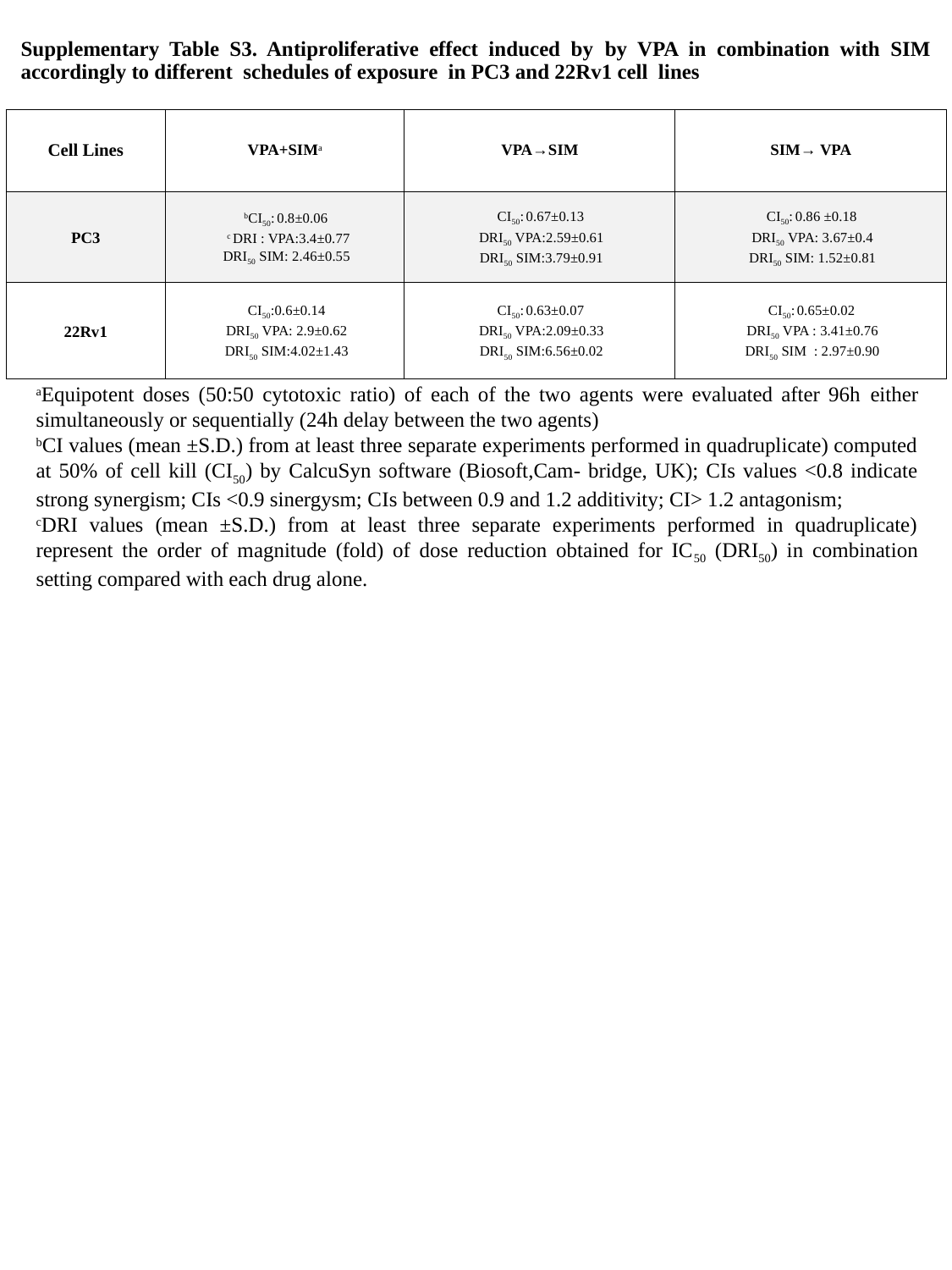

Supplementary Table S3. Antiproliferative effect induced by by VPA in combination with SIM accordingly to different schedules of exposure in PC3 and 22Rv1 cell lines
| Cell Lines | VPA+SIMa | VPA→SIM | SIM→ VPA |
| --- | --- | --- | --- |
| PC3 | bCI50: 0.8±0.06 c DRI : VPA:3.4±0.77 DRI50 SIM: 2.46±0.55 | CI50: 0.67±0.13 DRI50 VPA:2.59±0.61 DRI50 SIM:3.79±0.91 | CI50: 0.86 ±0.18 DRI50 VPA: 3.67±0.4 DRI50 SIM: 1.52±0.81 |
| 22Rv1 | CI50:0.6±0.14 DRI50 VPA: 2.9±0.62 DRI50 SIM:4.02±1.43 | CI50: 0.63±0.07 DRI50 VPA:2.09±0.33 DRI50 SIM:6.56±0.02 | CI50: 0.65±0.02 DRI50 VPA : 3.41±0.76 DRI50 SIM : 2.97±0.90 |
aEquipotent doses (50:50 cytotoxic ratio) of each of the two agents were evaluated after 96h either simultaneously or sequentially (24h delay between the two agents)
bCI values (mean ±S.D.) from at least three separate experiments performed in quadruplicate) computed at 50% of cell kill (CI50) by CalcuSyn software (Biosoft,Cam- bridge, UK); CIs values <0.8 indicate strong synergism; CIs <0.9 sinergysm; CIs between 0.9 and 1.2 additivity; CI> 1.2 antagonism;
cDRI values (mean ±S.D.) from at least three separate experiments performed in quadruplicate) represent the order of magnitude (fold) of dose reduction obtained for IC50 (DRI50) in combination setting compared with each drug alone.
