# Supplementary material for: Synergistic antitumor interaction of valproic acid and simvastatin sensitizes prostate cancer to docetaxel by targeting CSCs compartment via YAP inhibition
Source: J Exp Clin Cancer Res. 2020 Oct 8;39:213. doi: 10.1186/s13046-020-01723-7 (PMC7545949; doi:10.1186/s13046-020-01723-7)
Supplement: Supplementary file 3 — Additional file 3: Supplementary Figure S1. Soft agar clonogenic assay of DU145 (A) and DU145 R80 cells (B), untreated or treated with VPA and SIM alone and in combination at 1 mM and 0.625 μM respectively (IC2596h values) for both cell lines. Colonies of > 100 μm were scored by a colony counter. Right: images from a representative experiment; left: values are the mean ± S.D. from at least three independent experiments. Supplementary Figure 2. A. Characterization of the indicated prostate cancer cells for their ability to growth in low attach condition ad 3D-spheroids; all cell lines (40,000 cell/ml) were plated in low attach support and sphere medium for 72 h. B. Nanog (left panel) and OCT4(right panel) mRNA expression evaluated by RT-PCR at basal level in 22Rv1 in cell adhesion condition, in 1st generation spheres and in 2nd generation spheres. β-actin was used as housekeeping control gene to normalize RT-PCR reactions. C. Basal expression of NANOg evaluated by western blotting in 22Rv1 cells in cell adhesion condition, in 1st generation spheres and in 2nd generation spheres. Γtubulin was used as loading control. D. Surface marker expression (CD44 and CD113) was determined by flow cytometry on 22Rv1cells at basal level in 22Rv1 in both cell adhesion condition and 1st generation spheres. E. CTGF mRNA expression evaluated by RT-PCR at basal level in 22Rv1 in both cell adhesion condition and in 1st generation spheres. β-actin was used as housekeeping control gene to normalize RT-PCR reactions Statistically significant results are reported (*** indicates P < 0.0005, ** indicates P < 0.005 and * indicates P < 0.05). Supplementary Figure S3. A. Expression of acetyl histone H3 (AcH3) evaluated by western blot in the indicated cell lines, untreated or treated with VPA and SIM alone or in combination at the IC5096h for 24 h,; ponceau red serves as control for equal protein loading. B. HMGCR mRNA expression evaluated by RT-PCR in 22Rv1 cells untreated or treated for the ind [file 13046_2020_1723_MOESM3_ESM.ppt]

## Slide 1
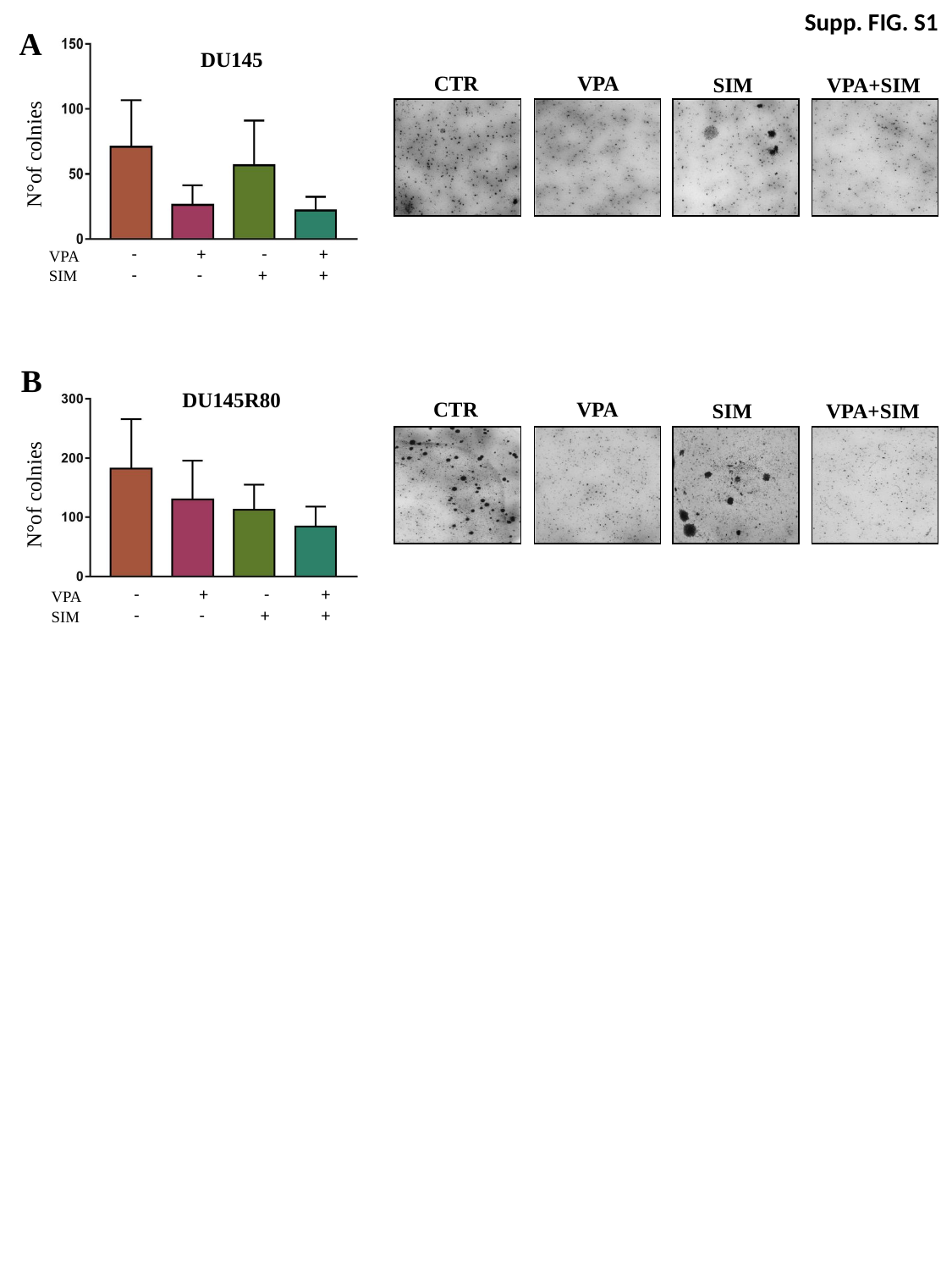

Supp. FIG. S1
A
DU145
CTR
VPA
SIM
VPA+SIM
N°of colnies
- + - +
VPA
SIM
- - + +
B
DU145R80
CTR
VPA
SIM
VPA+SIM
N°of colnies
- + - +
VPA
SIM
- - + +

## Slide 2
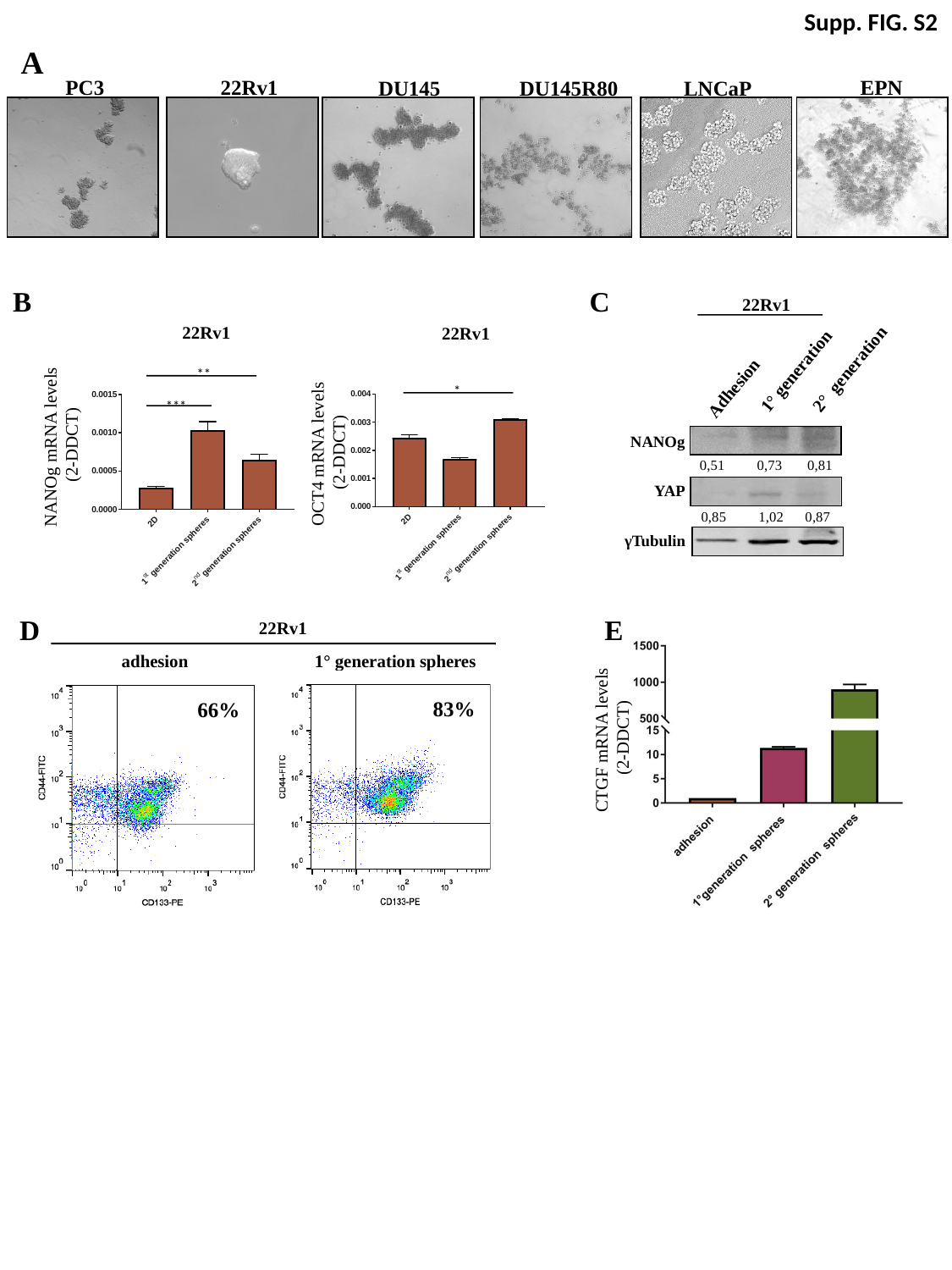

Supp. FIG. S2
A
PC3
22Rv1
EPN
DU145
DU145R80
LNCaP
B
C
22Rv1
22Rv1
22Rv1
2° generation
1° generation
**
Adhesion
*
***
NANOg mRNA levels
 (2-DDCT)
NANOg
OCT4 mRNA levels
 (2-DDCT)
0,51 0,73 0,81
YAP
0,85 1,02 0,87
γTubulin
D
E
22Rv1
adhesion
1° generation spheres
83%
66%
CTGF mRNA levels
 (2-DDCT)

## Slide 3
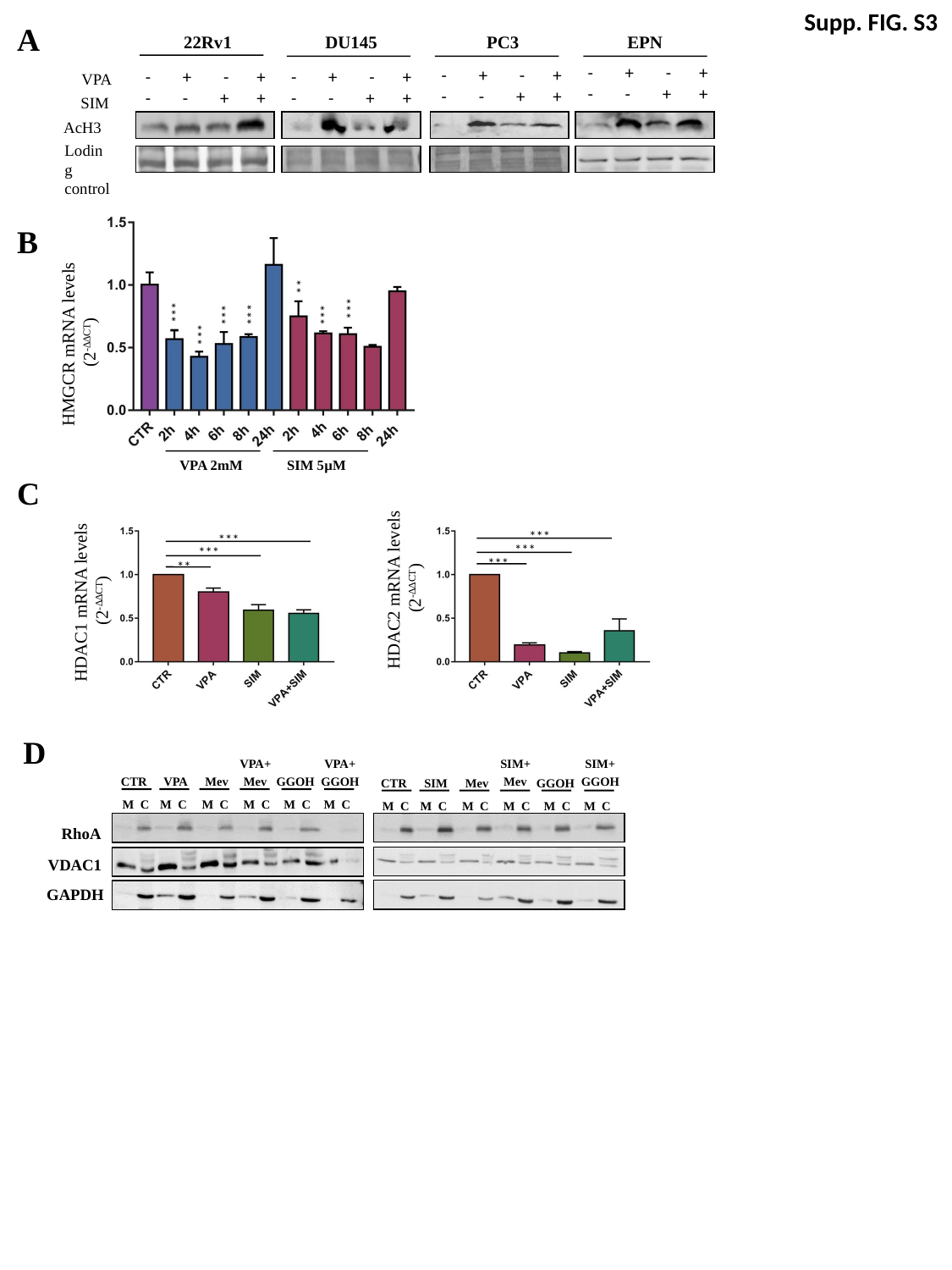

Supp. FIG. S3
A
22Rv1
DU145
PC3
EPN
- + - +
- + - +
- + - +
- + - +
VPA
- - + +
- - + +
- - + +
- - + +
SIM
AcH3
Loding
control
B
**
***
***
***
***
***
HMGCR mRNA levels
 (2-∆∆CT)
***
VPA 2mM
SIM 5µM
C
***
***
***
***
***
**
HDAC2 mRNA levels
 (2-∆∆CT)
HDAC1 mRNA levels
 (2-∆∆CT)
D
VPA+
Mev
VPA+
GGOH
SIM+
Mev
SIM+
GGOH
 CTR
VPA
Mev
GGOH
 CTR
SIM
Mev
GGOH
M C
M C
M C
M C
M C
M C
M C
M C
M C
M C
M C
M C
RhoA
VDAC1
GAPDH

## Slide 4
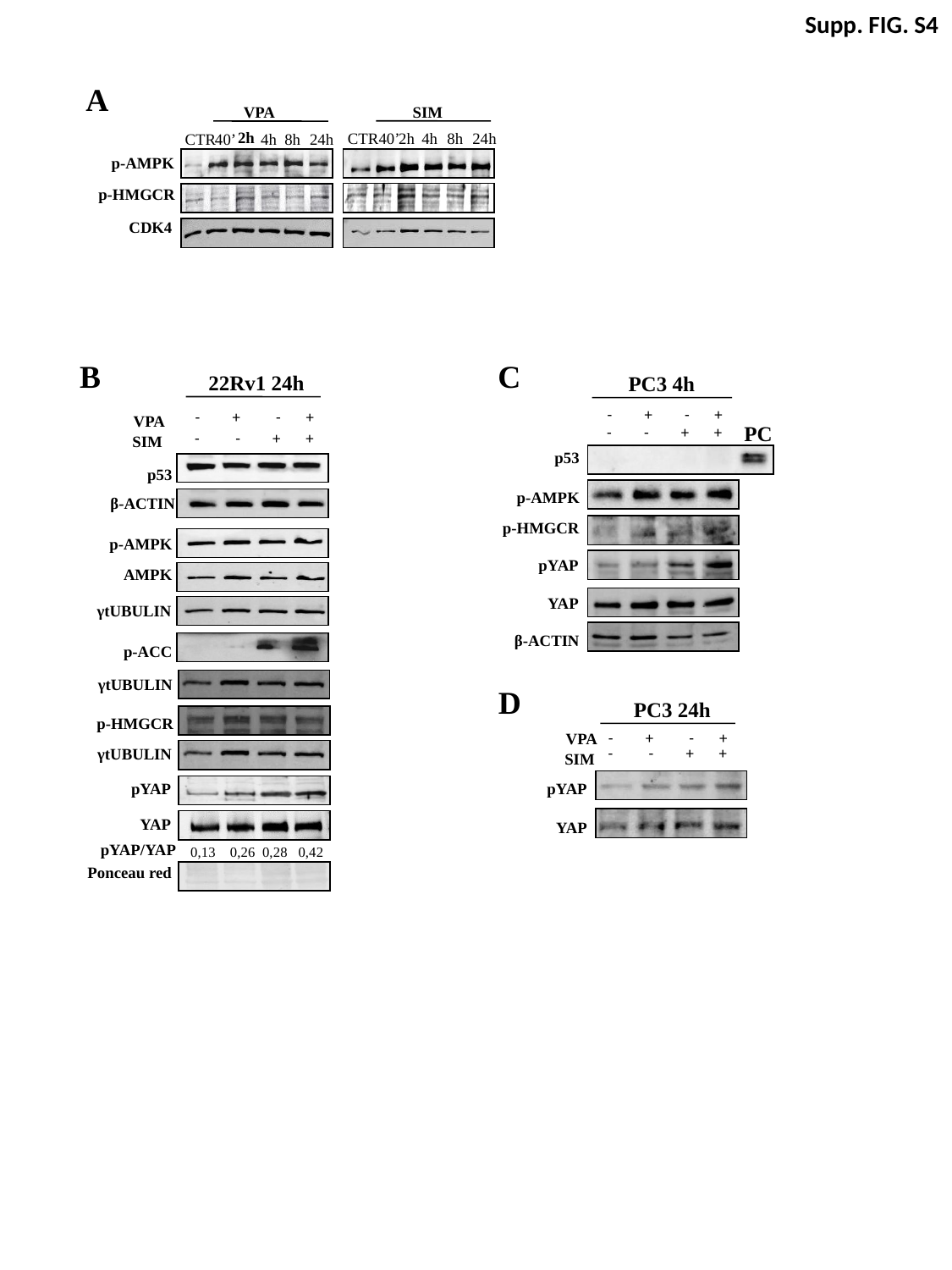

Supp. FIG. S4
A
VPA
SIM
CTR
40’
2h
4h
8h
24h
CTR
40’
4h
8h
24h
2h
p-AMPK
p-HMGCR
CDK4
B
C
22Rv1 24h
PC3 4h
- + - +
- + - +
VPA
- - + +
PC
- - + +
SIM
p53
p53
p-AMPK
β-ACTIN
p-HMGCR
p-AMPK
pYAP
AMPK
YAP
γtUBULIN
β-ACTIN
p-ACC
γtUBULIN
D
PC3 24h
p-HMGCR
- + - +
VPA
γtUBULIN
- - + +
SIM
pYAP
pYAP
YAP
YAP
pYAP/YAP
0,13 0,26 0,28 0,42
Ponceau red

## Slide 5
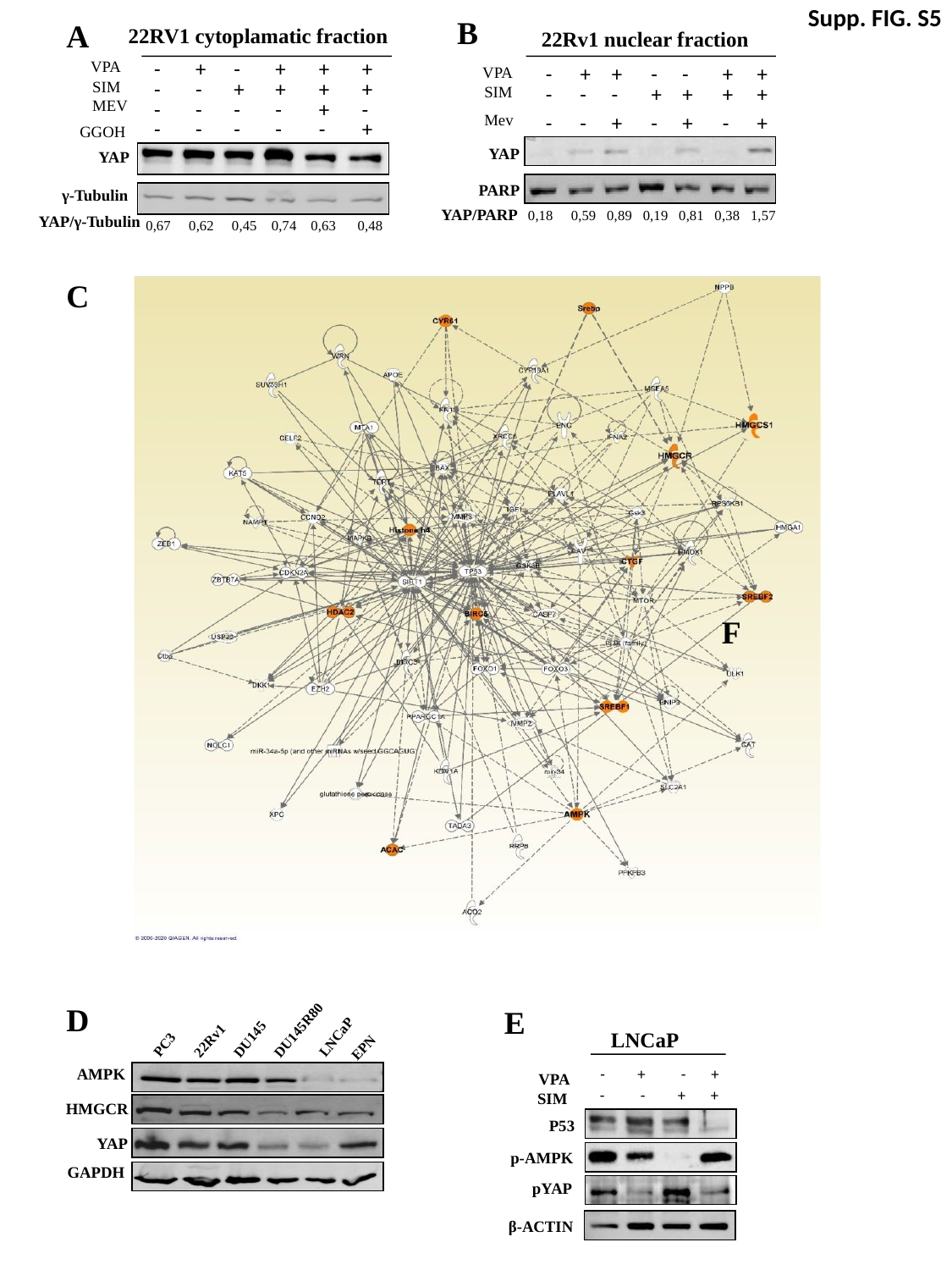

Supp. FIG. S5
B
A
22RV1 cytoplamatic fraction
22Rv1 nuclear fraction
VPA
-
+
-
+
+
+
VPA
-
+
+
-
-
+
+
SIM
-
-
+
+
+
+
SIM
-
-
-
+
+
+
+
MEV
-
-
-
-
+
-
Mev
-
-
+
-
+
-
+
-
-
-
-
-
+
GGOH
YAP
YAP
PARP
γ-Tubulin
YAP/PARP
0,18 0,59 0,89 0,19 0,81 0,38 1,57
YAP/γ-Tubulin
0,67 0,62 0,45 0,74 0,63 0,48
C
F
D
E
DU145R80
LNCaP
DU145
LNCaP
22Rv1
PC3
EPN
AMPK
- + - +
VPA
- - + +
SIM
HMGCR
P53
YAP
p-AMPK
GAPDH
pYAP
β-ACTIN

## Slide 6
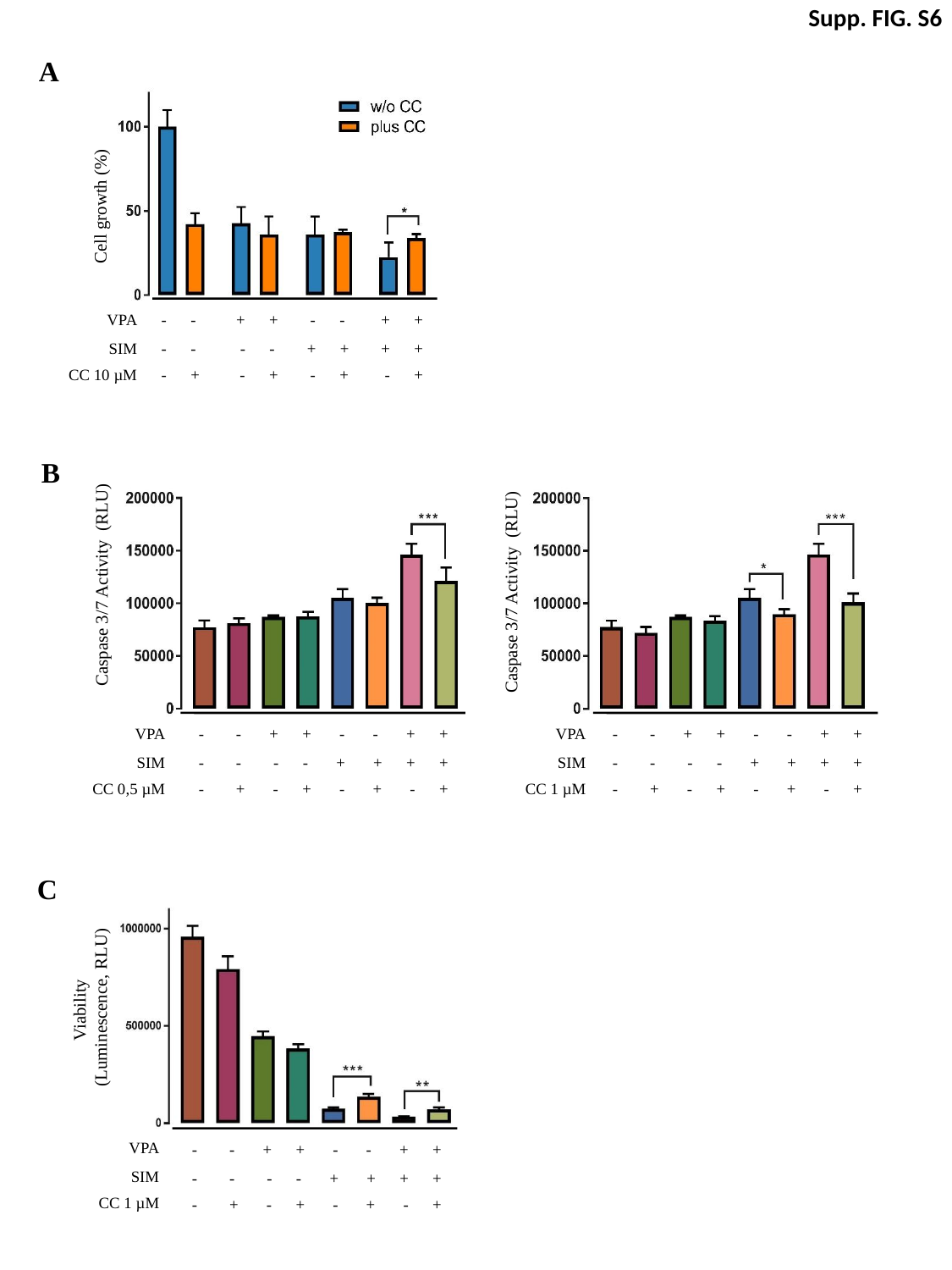

Supp. FIG. S6
A
Cell growth (%)
VPA
 - - + + - - + +
SIM
 - - - - + + + +
 CC 10 µM
 - + - + - + - +
B
Caspase 3/7 Activity (RLU)
Caspase 3/7 Activity (RLU)
VPA
 - - + + - - + +
VPA
 - - + + - - + +
SIM
 - - - - + + + +
SIM
 - - - - + + + +
 CC 0,5 µM
 - + - + - + - +
 CC 1 µM
 - + - + - + - +
C
Viability
(Luminescence, RLU)
VPA
 - - + + - - + +
SIM
 - - - - + + + +
 CC 1 µM
 - + - + - + - +

## Slide 7
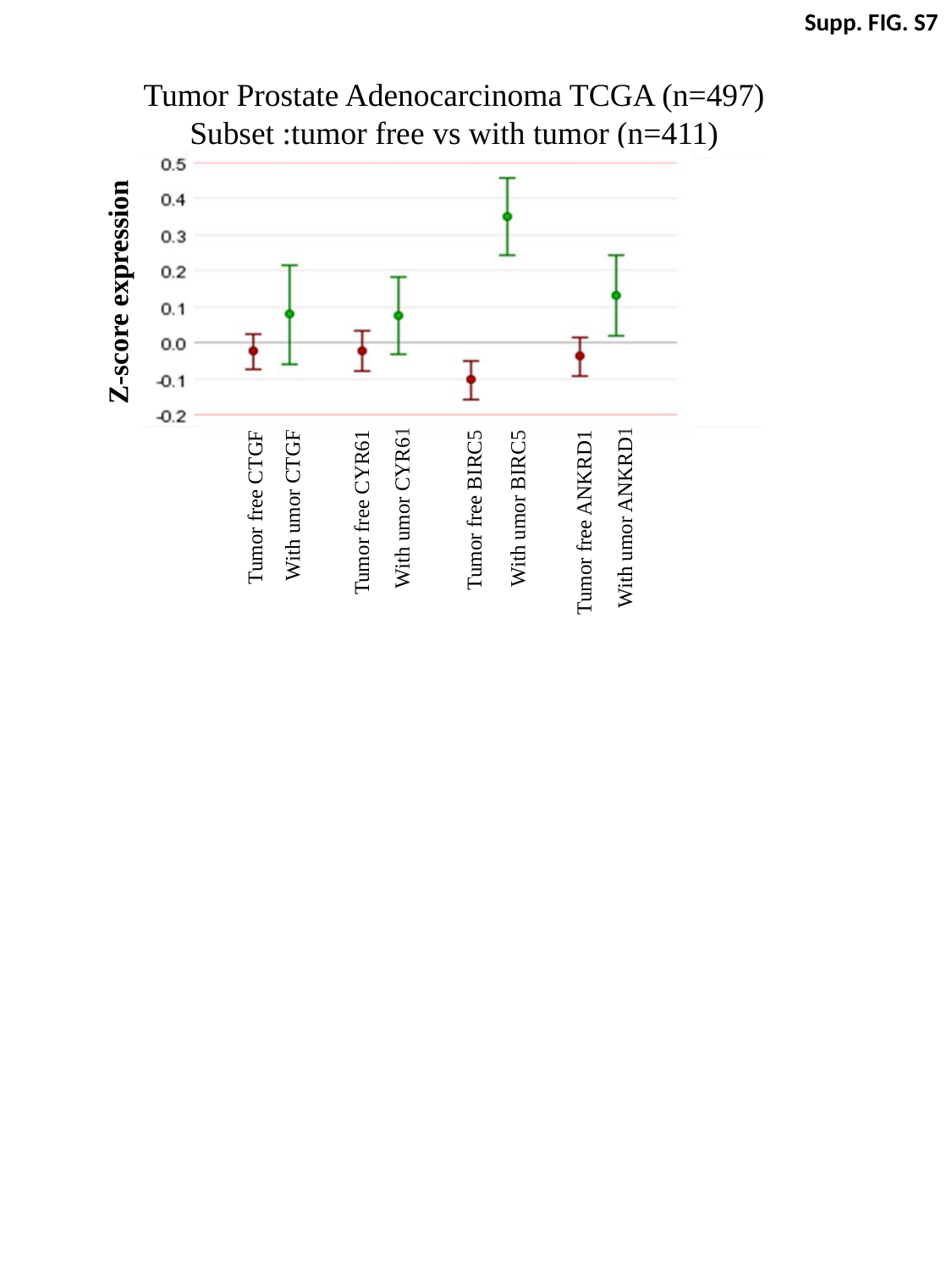

Supp. FIG. S7
Tumor Prostate Adenocarcinoma TCGA (n=497)
Subset :tumor free vs with tumor (n=411)
Z-score expression
With umor CTGF
Tumor free CTGF
With umor CYR61
With umor BIRC5
Tumor free BIRC5
Tumor free CYR61
With umor ANKRD1
Tumor free ANKRD1

## Slide 8
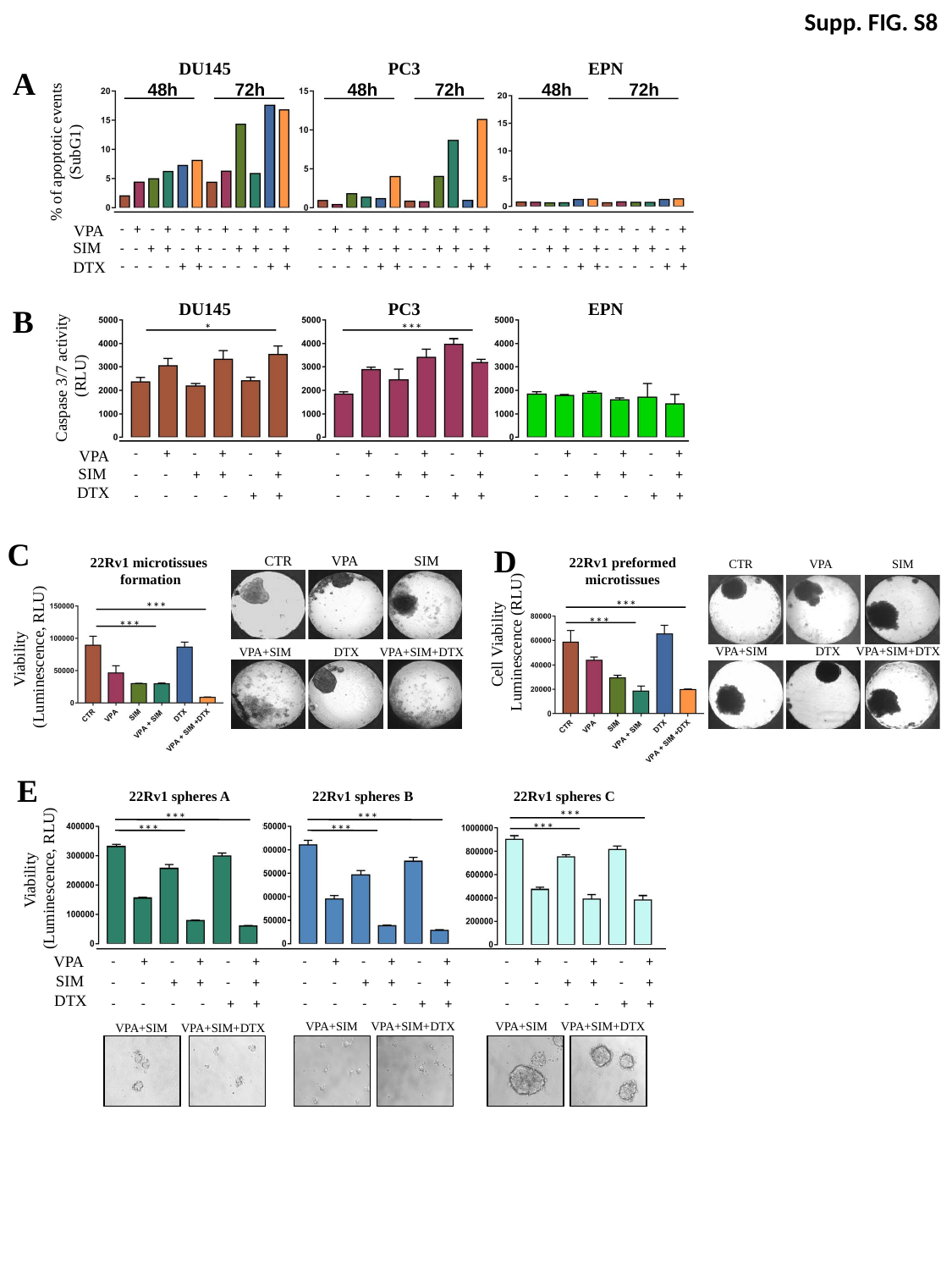

Supp. FIG. S8
DU145
PC3
EPN
A
48h
72h
48h
72h
48h
72h
% of apoptotic events
(SubG1)
VPA
- + - + - +
- + - + - +
- + - + - +
- + - + - +
- + - + - +
- + - + - +
SIM
- - + + - +
- - + + - +
- - + + - +
- - + + - +
- - + + - +
- - + + - +
DTX
- - - - + +
- - - - + +
- - - - + +
- - - - + +
- - - - + +
- - - - + +
DU145
PC3
EPN
B
*
***
Caspase 3/7 activity
 (RLU)
VPA
- + - + - +
- + - + - +
- + - + - +
SIM
- - + + - +
- - + + - +
- - + + - +
DTX
- - - - + +
- - - - + +
- - - - + +
C
D
CTR
VPA
SIM
22Rv1 microtissues
 formation
22Rv1 preformed
microtissues
CTR
VPA
SIM
***
***
***
***
Cell Viability
Luminescence (RLU)
Viability
(Luminescence, RLU)
VPA+SIM
DTX
VPA+SIM+DTX
VPA+SIM
DTX
VPA+SIM+DTX
E
22Rv1 spheres A
22Rv1 spheres B
22Rv1 spheres C
***
***
***
***
***
***
Viability
(Luminescence, RLU)
VPA
- + - + - +
- + - + - +
- + - + - +
SIM
- - + + - +
- - + + - +
- - + + - +
DTX
- - - - + +
- - - - + +
- - - - + +
VPA+SIM
VPA+SIM+DTX
VPA+SIM
VPA+SIM+DTX
VPA+SIM
VPA+SIM+DTX

## Slide 9
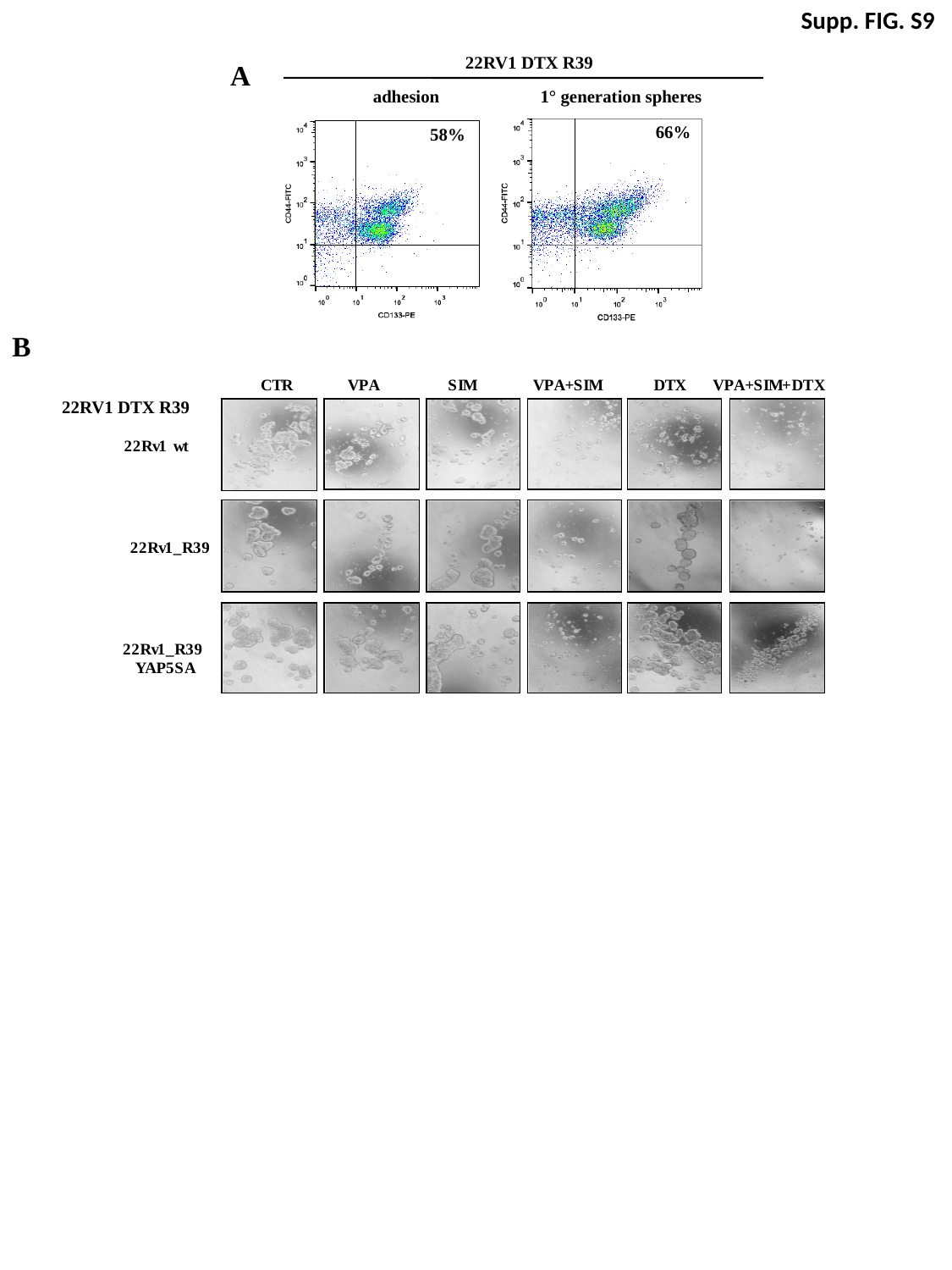

Supp. FIG. S9
22RV1 DTX R39
A
adhesion
1° generation spheres
66%
58%
B
22RV1 DTX R39

## Slide 10
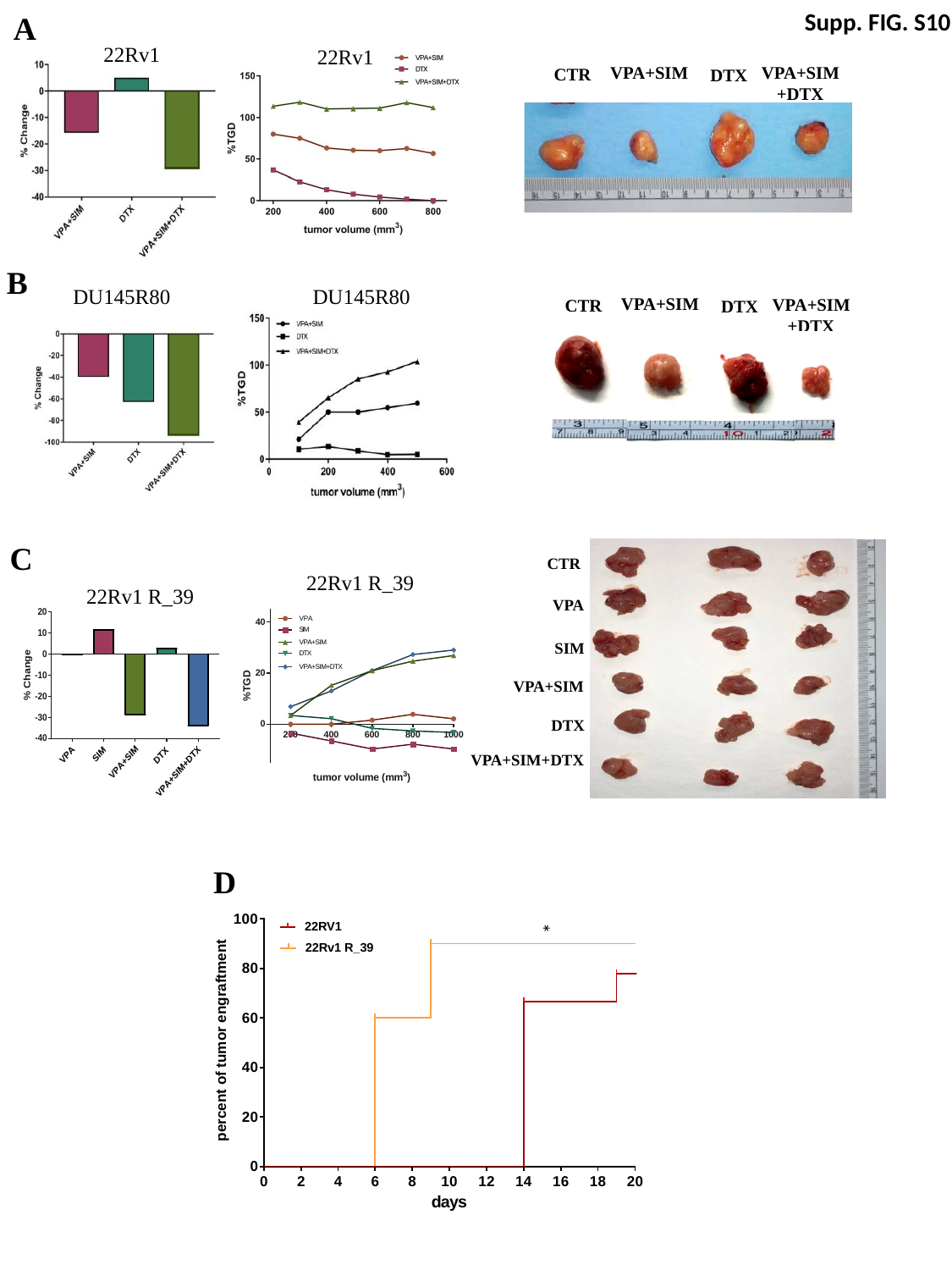

Supp. FIG. S10
A
22Rv1
22Rv1
VPA+SIM
VPA+SIM
+DTX
CTR
DTX
B
DU145R80
DU145R80
VPA+SIM
VPA+SIM
+DTX
CTR
DTX
C
CTR
22Rv1 R_39
22Rv1 R_39
VPA
SIM
VPA+SIM
DTX
VPA+SIM+DTX
D
*
